# Advanced Nanomaterials Functionalized with Metal Complexes for Cancer Therapy: From Drug Loading to Targeted Cellular Response

**DOI:** 10.3390/ph18070999

**Published:** 2025-07-03

**Authors:** Bojana B. Zmejkovski, Nebojša Đ. Pantelić, Goran N. Kaluđerović

**Affiliations:** 1 Department of Chemistry, Institute of Chemistry, Technology and Metallurgy, National Institute of the Republic of Serbia, University of Belgrade, Studenski trg 12-16, 11000 Belgrade, Serbia; 2 Department of Chemistry and Biochemistry, Faculty of Agriculture, University of Belgrade, Nemanjina 6, 11080 Belgrade, Serbia; pantelic@agrif.bg.ac.rs; 3 Department of Engineering and Natural Sciences, University of Applied Sciences Merseburg, Eberhard-Leibnitz-Straße 2, DE-06217 Merseburg, Germany

**Keywords:** nanomaterials, drug delivery, metal complexes, theranostics, biomedical application, anticancer activity

## Abstract

Developments of nanostructured materials have a significant impact in various areas, such as energy technology and biomedical use. Examples include solar cells, energy management, environmental control, bioprobes, tissue engineering, biological marking, cancer diagnosis, therapy, and drug delivery. Currently, researchers are designing multifunctional nanodrugs that combine in vivo imaging (using fluorescent nanomaterials) with targeted drug delivery, aiming to maximize therapeutic efficacy while minimizing toxicity. These fascinating nanoscale “magic bullets” should be available in the near future. Inorganic nanovehicles are flexible carriers to deliver drugs to their biological targets. Most commonly, mesoporous nanostructured silica, carbon nanotubes, gold, and iron oxide nanoparticles have been thoroughly studied in recent years. Opposite to polymeric and lipid nanostructured materials, inorganic nanomaterial drug carriers are unique because they have shown astonishing theranostic (therapy and diagnostics) effects, expressing an undeniable part of future use in medicine. This review summarizes research from development to the most recent discoveries in the field of nanostructured materials and their applications in drug delivery, including promising metal-based complexes, platinum, palladium, ruthenium, titanium, and tin, to tumor cells and possible use in theranostics.

## 1. Introduction

Specific demands in biomedicine and engineering have driven the development of novel materials with tailored properties. Rapid advances in nanoscience support the creation of nanomaterials and nanotechnologies aimed at solving complex medical challenges. Modern medicine increasingly requires multifunctional systems capable of simultaneous imaging and drug delivery. Nanostructured materials’ surface functionalization is also of great importance. Functional groups must be placed on the nanomaterial surfaces so they can conjugate biomolecules for targeting, virus detection, or drug delivery. The World Health Organization is predicting an increasing number of cancer-related deaths, partly caused by the global aging of the population. Cancer is the cause of death of nearly 10 million people worldwide, and there were over 18 million new cases reported in 2020 [1], and comparable figures observed in 2024 [2], indicating a continuous upward trend in cancer incidence and mortality [3]. Cancer, as a leading cause of death, is also influenced by lower mortality rates due to stroke and heart disease in many countries [1].

Cancer cells are subtly mutated versions of normal cells, capable of uncontrolled proliferation. This similarity makes it challenging to develop therapies that selectively target cancer cells without affecting healthy ones [4,5]. The three well-known main ways to battle cancer, surgery, radiotherapy, and chemotherapy, developed over recent years are clearly giving patients more benefits due to progress in surgery and advanced chemotherapeutic treatment. However, overall outcomes show only marginal improvement, as most late-stage cancers remain a significant therapeutic challenge. In addition, the usual drawbacks—selectivity, dose-limiting toxicity, resistance, and patients experiencing severe side effects [6,7]—have stimulated major research efforts. Because of this, new approaches and re-evaluation of diagnostic tools and treatment procedures are urgently needed to improve today’s methods. Nanomedicine holds great promise, particularly through the use of nanoparticles (NPs) as advanced engineering tools. Using nanostructured systems to deliver anticancer metallodrugs is a developing field of research. In the literature, a number of reviews thoroughly address the way to apply nanomaterials in drug delivery, regenerative medicine, tissue engineering, molecular imaging, and anticancer therapy [8,9,10,11,12,13,14,15,16,17,18,19].

This review aims to provide an overview of recent advancements in the functionalization of nanostructured materials with metallodrugs (platinum and non-platinum, classical coordination, and organometallic anticancer drugs) as future inventive alternatives to safer and efficient anticancer therapy.

## 2. Nanomaterials

Nanostructured materials have a classification based on the dimensions of their basic structural elements, as the prefix “nano” refers to “one billionth” (10^−9^) of a meter. Regarding the drug delivery nanoscale systems discussion, all nanomaterials (NMs) could be called “nanoparticles” or, in short, “NPs”. The rapidly advancing nanotechnology has a striking influence on cancer therapy and diagnosis, especially nanoparticles [20,21,22], which can enable targeted delivery of anticancer drugs. These particles typically range from 1 to 100 nm in diameter, although larger sizes are also encountered, as nanotechnology does not strictly define size boundaries [23,24,25]. Particles this size have different physical, mechanical, electrical, and optical properties than those observed in the macroscopic and atomic domains [26,27,28].

Drug delivery systems should overcome limitations of current therapeutics, such as low stability and potential immunogenicity, thereby enhancing efficacy while reducing typical toxic side effects [14,25,29]. Furthermore, despite poorer drug loading capacities, the nanometer size enables the drug carriers to cross the cell membranes, reducing unwanted elimination from the body [14,25,29,30].

Up to date, drug delivery is mostly achieved using liposomal, polymeric, and inorganic carriers [14,31,32,33]. In nanoscale proportions, liposomes possess a very high capacity to carry drugs, but how they release them is hard to regulate. On the other hand, polymeric systems can be designed to generate desired molecular weights and structure, although their capacity to carry drugs is, in relevance, low [14,34]. Inorganic carriers possess unique optical, electronic, and magnetic properties, as well as porous mesostructures with high surface area, tunable pore sizes, and easily modifiable surfaces, all of which make them promising candidates for theranostic applications [14,35,36].

## 3. Nanodrug Delivery System

Drug delivery is, without a doubt, one of the most meaningful advantages given by the use of nanotechnology. Properties of chemotherapeutics used regularly are improved by delivering them to their biological targets [37,38]. Nanotechnology can enhance even drugs that previously failed in clinical trials. Additionally, it can improve the solubility of poorly soluble drugs, allowing for better administration [39,40,41,42]. Merging them with lipid or organic NMs may keep them circulating longer and increase stability [43,44,45]. Moreover, many drugs’ main issue is crossing the blood–brain barrier. Nanoparticles loaded with anticancer drugs have the possibility to penetrate the mentioned barrier and help maintain high concentrations of them in brain tumors [46,47,48].

Nanoscale devices are in the size range of large biomolecules—enzymes and receptors—which are used for “active” drug delivery when a nanoparticle is functionalized with molecules for selective binding to moieties specific for tumors revealed at the target cells [48,49,50,51]. These moieties include receptors, transporters, or antigens of which quantity or functionality is fairly higher in tumors in comparison to normal cells.

Nanoscale devices can easily enter most cells. This feature is used in “passive” targeting of antitumor agents, which is more closely based on an enhanced permeability and retention, shortly called the EPR effect [52]. This effect happens due to the fact that solid tumors possess a leaky vasculature joined with the absence or reduced lymphatic drainage, which is the reason for high molecular weight molecules (polymers) accumulation.

### 3.1. Passive Accumulation in Solid Tumors

Drug-delivery systems based on nanocarriers (so-called passive targeting) include polymers [53], micelles [54,55], liposomes [56], dendrimers [57], silica nanoparticles [58], and organic/inorganic hybrid NMs [59]. They can be used as carriers for the tumor-specific delivery of biologically active molecules—“BAMs” (drugs)—by encapsulating or covalently binding the low molecular weight (LMW) molecule to the carrier. When the encapsulated drug reaches the tumor because of the EPR effect [60], the drug should be released.

It is possible to create nanostructured materials providing controlled release of antitumor drugs and keep drugs inside until a specific signal to release is provided, so-called “zero release until NMs reach the tumor”.

Controlled drug release in tumors can be triggered by various stimuli, such as acidic pH in the tumor microenvironment, hydrolysis inside cancer cells, enzyme-mediated breakdown in lysosomes (enzymolysis), redox reactions in the cytosol, or external factors like light and magnetic fields. Besides delivering anticancer drugs, a fluorescent dye can be carried and enable imaging. Thus, diagnostics and therapeutics (theranostics) can be carried out simultaneously [61,62]. It is expected that this emerging field of medicine employing multifunctional nanomaterials is going to be a real cancer therapy in the near future. Moreover, it is expected that such NMs will likely bypass some of the undesirable biological effects.

The first FDA-approved nanodrug was a PEGylated liposomal formulation of doxorubicin (DOX), used to treat Kaposi’s sarcoma and ovarian cancer. Encapsulation significantly reduced systemic toxicity, improved pharmacokinetics, and enabled controlled drug release [63,64]. More recently, a liposomal combination of cytarabine and daunorubicin (CPX-351, Vyxeos™) was approved and demonstrated a median survival of 9.6 months, compared to 6 months for the free drug form in high-risk acute myeloid leukemia patients [65]. There are more formulations that have been approved [63,66,67,68], pointing out new therapeutic routes to fight cancer.

### 3.2. Active Targeting Ligands

Since the EPR effect is non-selective, relying solely on it can undermine the precision of active targeting strategies. Moreover, the word “targeting” should apply only to “active targeting”, which is based on a membrane receptor interaction with a 3D-matched ligand tied up to the surface of the drug delivery system, where its role is targeting [69,70,71,72].

The idea of a drug being a magic bullet that finds the biological target in the body is matched to units leading drug warheads to cancer cells by navigated interactions with receptors overexpressed on the surface [5,60,67,73,74]. This idea can become more comprehensive in accomplishing targeting the whole tumor, not only cancer cells, by using selective activation in the tumor microenvironment, which is hypoxic or acidic, or using proteins expressed on angiogenic blood vessels [75]. Finally, targeting might take place at the subcellular level, meaning a drug can be targeted to specific organelles.

#### 3.2.1. Sugar Targeting

Sugar biology may be a good concept for drug targeting, simply because of the increased uptake of glucose by cancer cells [76]. It is known that the multiplication of cells at such a large level requires larger amounts of nutrients, especially glucose [77].

Platinum complexes have the potential to bind to various sugars. Still, there is not much evidence to suggest that sugars, including glucose, have amplified the action of the anticancer drug by attachment to a specific receptor [78].

Aerobic glycolysis, a known property of cancer cells, is also a property of immune and stem cells. If a therapeutic agent cannot distinguish between malignant and nonmalignant cells, targeting metabolic pathways may result in toxicity similar to that of conventional chemotherapeutics [79].

#### 3.2.2. Steroid Targeting

Testosterone and estrogen, sex hormones, can be of high significance in drug targeting. Estrogen receptors (ERs) are highly expressed on the surfaces of some cancer cells, and it is well known in breast cancer and may be used as a drug target.

The anticancer activity of platinum compounds can be modulated through their interaction with estrogen receptors (ERs), either by disrupting receptor function or by enhancing cellular uptake. This mechanism increases the likelihood of DNA platination and promotes apoptotic cell death. The same stands for testosterone-expressing androgen receptors (ARs) to target platinum to those cancer cells [78]. Transport proteins that uptake bile salt from blood are expressed on the hepatic epithelial cells; thus, for the purpose of delivering drugs directly to the liver, platinum complexes have been used for interacting with bile acids as they are also steroids [78].

#### 3.2.3. Peptide Targeting

Conjugation of platinum(II) complexes to a peptide result in formation of cyclic peptide c(CNRGC), which includes the Asn–Gly–Arg sequence. This conjugate targets the CD13 receptor on the surface of specific cancer cells [80] and demonstrates greater toxicity against CD13-expressing prostate cancer cells than carboplatin [78,81].

#### 3.2.4. Folic Acid Targeting

A significant variety of cancer cell lines and tumors taken from patients show overexpression of a glycoprotein that plays a role as a folate receptor (FR) [82].

The reason is that there is a pteroic acid unit that folic acid contains, which is of crucial importance to the main biochemical pathways in the body, for example, those connected to DNA synthesis. Again, the cell division of cancer cells, which is uncontrollable, needs more nutrients; thus, folate uptake is enhanced. Folate motif may be used to target platinum complexes due to their role in DNA synthesis and the increased folate uptake by rapidly dividing cancer cells [83]. However, studies have shown that when cisplatin interacts with folates, chloride ligands are replaced by tetrahydrofolate, forming a stable complex with limited cytotoxicity due to the absence of labile coordination sites [84]. The first approved BiTE by the FDA in 2014—a BiTE named blinatumomab (Blincyto^®^)—was intended for subgroups of leukemia patients. Numerous cancer antigens were after used as targets for bispecific antibodies [85,86,87].

## 4. Functionalization of Nanosized Materials with Metallodrugs

Research developments ensure optimum loading and delayed release of metallodrugs and that they are attached to the surface and/or stored in an NM with enough strength to reach the target site but, at the same time, weak enough to release upon delivery.

Principally, functionalization of nanomaterials with metallodrugs, prodrugs, or contrast agents is performed by tethering to surface ligands (onto NMs) or purely by encapsulation (into NMs). Countless approaches were designed to create connections between metallodrugs, biomolecules, and NMs [88,89,90,91,92]. Cross-linking between amines and carboxylic acids, typically achieved via carbodiimide activation, is commonly used. However, this method is prone to side reactions, which can limit its applicability.

Another commonly used drug conjugation policy is the reaction of maleimides with thiols (usually cysteine residues of proteins). The coupling of maleimides with thiols is extremely specific, resisting hydrolysis, and can take place at neutral pH with no uninvited side reactions [93].

Click chemistry reactions, for instance [3 + 2] cycloaddition reactions of azides and alkynes, yield a triazole linkage. The mentioned method was correspondingly applied to conjugate NMs to metal complexes [94,95].

Diels–Alder cycloaddition reactions with reverse electron demand occur, and these bioconjugations happen easily and quickly at normal temperature under physiological conditions with no need for a catalyst [96,97]. Indeed, different reactions were applied less for conjugation reasons [98,99]. Additionally, metallodrugs may always be functionalized onto/into nanostructures via covalent or hydrogen bonding and hydrophobic and electrostatic interactions, enabling more accessible drug release when the target site is reached. But first, it is proper to discuss what types of nanomaterials are used.

### 4.1. Liposomes and Their Drug Formulations

**Figure 1:**
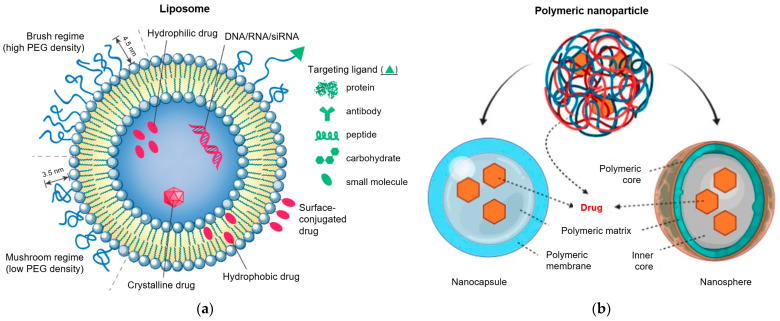
(**a**) Schematic representation of liposomal drug delivery [103]; (**b**) schematic representation of polymeric nanoparticles [104].

Phospholipids and related molecules are commonly used to prepare liposomal compositions, which may also contain small amounts of other components. Liposome sizes range from a few nanometers to tens of micrometers. Smaller unilamellar liposomes often have targeting ligands on their surface, allowing them to accumulate in pathological areas. [100]. Liposomes are classified into multilamellar vesicles (MLVs), small unilamellar vesicles (SUVs), large unilamellar vesicles (LUVs), and cochleate vesicles [101]. Many liposomal drug formulations are going through clinical trials, and several have been approved [102]. The major advantages are the possibility of using hydrophobic and hydrophilic drugs: the first ones can be enclosed in the phospholipid bilayers, and the second ones can be entrapped within their aqueous cavity [44]. In addition, liposome size, surface properties, and charge may be managed as needed (Figure 1a) [44,103].

To increase circulation time and avoid clearance by the immune system, stealth liposomes have been designed with polyethylene glycol (PEG) on their surface. The terminal PEG groups can also be modified to allow conjugation with various targeting ligands (Figure 1a).

### 4.2. Polymeric Nanoparticles

Biodegradable and biologically compatible polymers are in use to prepare polymeric nanoparticles (PNPs) with different sizes. An active agent or drug can be dissolved, encapsulated, or attached to the matrix of the nanoparticle. Nanospheres and nanocapsules may be acquired according to the synthetic method used [105].

Both of them may carry a drug, whereas in nanocapsules, drug cargo is in a cavity entrapped by a polymer membrane, and in nanospheres, the drug is uniformly and physically dispersed in a matrix system (Figure 1b) [104]. The nanotechnology of polymer nanoparticles (PNPs) is rapidly growing to an extensive range of areas—conducting materials, electronics, photonics, sensors, biotechnology, medicine, environmental technology, pollution control, etc. [105].

Polymer nanoparticles are convenient vehicles that are gladly used in drug delivery to a specific target due to the easy management of their properties [106]. Their advantage is also increasing drug safety. PNPs could securely carry proteins, drugs, and DNA to biological target cells or organs. Possessing nano-size enables easy infiltration through the membranes of cells and provides high stability in the bloodstream [105,107]. Two types of polymers that can be applied in nanodelivery are natural and synthetic.

Natural: chitosan, cellulose, xanthan gum, starch, alginates, carrageenan, gellan gum, pectins. Synthetic: poly(lactic acid) (PLA), poly(cyanoacrylates) (PACA), poly(anhydrides), poly(acrylic acid), poly(ortho esters), poly(amides), poly(vinyl alcohol) (PVA), poly(ethylene glycol), and other like poly(ethylene oxide) (PEO), poly(isobutylcynoacrylate) (PIBCA), poly(ethylene oxide) (PEO), etc. [105].

Modern polymer prodrugs typically consist of a water-soluble polymer, a biologically active molecule (such as a low molecular weight drug), and a biodegradable spacer to enable controlled release [69]. In most cases, a fourth component is also present, which gives multivalence to the polymer carrier: a targeting moiety that provides specific delivery to target cells. The polymer prodrug can also be decorated/modified to have an additional active molecule. Conjugates with anticancer drugs or, more importantly, radionuclides present a hopeful group called immunotherapeutics and radioimmunotherapeutics for effective and precise treatment or diagnosis of cancer. In novel therapeutics, α emitters such as ^223^Ra, ^230^U, ^213^Bi, ^149^Tb, or β emitters ^60^Co, ^131^I, ^90^Y, accomplished in damaging DNA, are joined with mAbs possessing harmful effects against cancer cells by themselves [69]. A large variety of drug conjugates and polymer-carriers are being examined, and research is expanding; therefore, promising results are reviewed extensively [108,109,110,111,112,113,114,115].

### 4.3. Dendrimers

Globular in shape, dendrimers are monodisperse, while being well-defined, vastly bifurcated, 3D structures. Functionalization of the surface is effortless in a controlled manner. These outstanding nanostructures seem made to be applicants for being drug delivery systems [116,117]. Drugs are loaded in dendrimers in three different ways: (1) they are physically encapsulated, (2) they have electrostatic interactions, or (3) using covalent bonding conjugations. Because of the dense, globular structure, accessibility of inner cavity spaces, and numerous surface functional groups, active drugs could be encapsulated in the interior of the dendrimers, so-called physical encapsulation, and also attached to surface functional moieties, named covalent conjugations [118].

**Figure 2:**
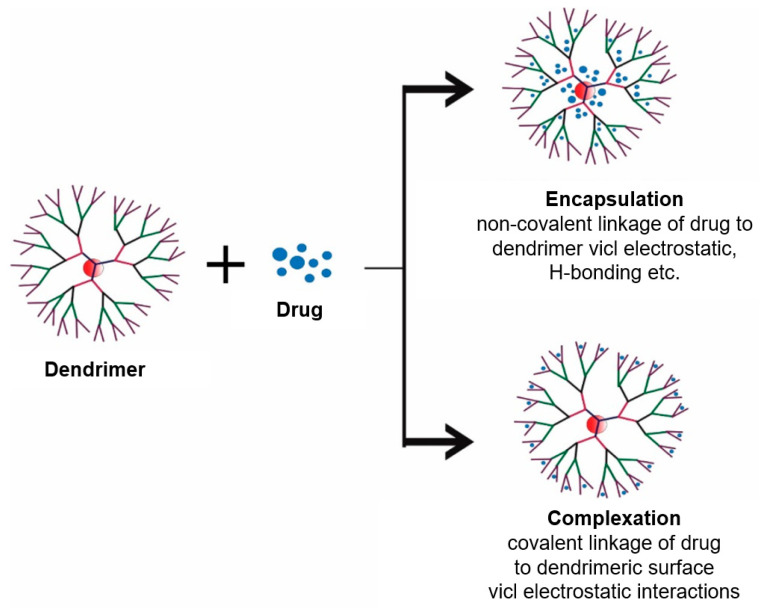
Drug loading in dendrimers. Simple encapsulation, electrostatic interaction, and covalent bonding conjugation [121]. Copyright 2019 Bentham Science Publishers.

PAMAM poly(amidoamine) is the most examined one for oral drug use because of its solubility in water. Functionalization groups divide dendrimers into several types. Nevertheless, the possibility of passing through epithelial tissue is enhancing the transfer by the paracellular pathway [119]. Dendrimers are often modified to eliminate toxicity due to the positively charged amine groups they possess. This limits their clinical use. In dendrimers, as represented in Figure 2, drug loading is achieved by encapsulation, electrostatic interaction, and covalent bonding conjugation [120,121].

Dendrimer-based drug delivery can follow two main pathways: (1) enzymatic cleavage of the drug–dendrimer bond in vivo, and (2) drug release in response to environmental factors such as temperature or pH [120]. Dendrimers develop in many areas, including pulmonary, targeted drug delivery, ocular, oral, and transdermal [122].

Seen as miracle carriers, dendrimers are being researched for serious illnesses, like cancer, tuberculosis, and even AIDS. In addition, attributed to their easy synthesis, great drug loading capacity, transdermal capability, stability, and oral drug-delivery potential, dendrimers were examined for controlled delivery of numerous anticancer drugs [118].

Dendrimeric drug delivery is fixated on chemotherapeutic bioactive agents, including cisplatin [123], methotrexate [124], and 5-fluorouracil [125], having slow release, better accumulating in solid tumors, and reduced toxicity compared to free drugs [126,127].

### 4.4. Carbon Nanotubes

**Figure 3:**
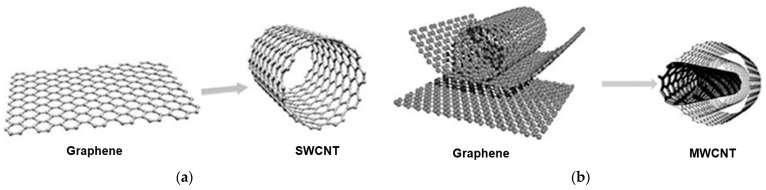
Representation of graphene and carbon nanotubes as (**a**) single-walled carbon nanotube (SWCNT) and (**b**) multi-walled carbon nanotube (MWCNT) structures [129].

Nanotubes are considered one-dimensional (1D) analogs of zero-dimensional (0D) fullerenes. They possess a hollow cylindrical structure formed by one-atom-thick graphene sheets rolled at a specific angle [128]. Nanotubes are categorized as single-walled nanotubes (SWCNTs), Figure 3a, and multi-walled nanotubes (MWCNTs), Figure 3b [129]. Individual nanotubes obviously align into “ropes” held by van der Waals forces, actually π–π stacking.

Since their discovery in 1991 by Ijima [130], single-walled carbon nanotubes have provoked much research in laboratories and industry. Speaking of biomedical applications, these remarkable materials initiate investigations toward finding imaging agents and tumor targeting [131].

Nanotubes are nowadays used for the delivery of metallodrugs. These materials enhance drug bioavailability over a longer time and improve solubility. Cisplatin was used for functionalization of SWNHs by Ajima et al. [132]. Additionally, platinum(IV) complexes having carboxylic acid (COOH) groups were conjugated to amine-functionalized single-walled carbon nanotubes (SWCNTs) via peptide bonds. These functionalized nanotubes exhibited enhanced cellular uptake and higher intracellular concentrations compared to free platinum(IV) complexes and cisplatin [133]. Research was carried out with different platinum complexes using various carbon nanostructures [134].

Carbon nanotubes are one of the most intriguing NPs used as theranostic tools, since they possess unmatched elasticity and conductivity [135,136]. A high loading capacity of theranostic agents might be achieved in NPs. Such drug-loaded NPs are found, through in vitro and in vivo studies, to be biocompatible. Lippard and coworkers reported the immobilization of cisplatin(IV) prodrugs in single-walled carbon nanotubes (SWCNTs). SWCNT NPs containing cisplatin analogues were water soluble and efficiently endocytosed by testicular cancer cells. Consequently, fast cargo (cisplatin) discharging from the NPs could be demonstrated due to intracellular reduction. In comparison to the drug itself, the efficiency was boosted by more than 100 times [133]. Similarly, multi-walled carbon nanotubes (MWCNTs) were used as a carrier (through hydrophobic interactions) of cisplatin(IV) prodrug. Inert and strongly hydrophobic platinum(IV) complexes are produced from appropriate platinum(II) species by reduction, which are formed analogously to cisplatin mono- and bifunctional adducts with deoxyguanosine monophosphate [137]. Carbon nanomaterials, as platinum drug-delivering systems, could make a broad choice of innovative cancer drugs available, for example, in photoacoustic, photothermal, and radiofrequency therapy. Multifunctional NPs, in combination with nucleotides and other anticancer drugs, are intended to be evolved to overcome the devastating drug resistance problem in current cancer chemotherapy and ultimately enhance tumor treatment efficacy.

Again, inorganic nanoparticles, for example, nanodiamonds, gold nanoparticles, and mesoporous silica nanostructured materials, are in use for the purpose of drug delivery [138]. Lastly, naturally engineered products, including even modified virus particles, were designed [139].

### 4.5. Nanodiamonds

Diamond nanoparticles of approximately 5 nm in size propose tremendous and changeable surface chemistry [140]. They have unique thermal and optical properties and exert no toxicity. The prospective of nanodiamonds in drug delivery was shown, even though the basic mechanisms of drug adsorption on nanodiamonds are not entirely clear. The most meaningful factors are purity, dispersion value, the chemistry of their surface, dispersion value, ionic composition, and temperature.

Extraordinary properties of nanodiamonds having attached molecules are that they can penetrate the blood–brain barrier. For example, in 2013, doxorubicin (DOX) molecules (one of the clinically used drugs) were bonded to the surfaces of nanodiamonds, creating the drug ND-DOX. As a result, tumors could not excrete the compound that was revealed by multiple tests, increasing the capability of the drug to affect the tumor while also decreasing the side effects [141].

### 4.6. Metal and Different Magnetic Materials NPs

The assets of metal nanostructured materials are very handy for imaging and therapy in medicine. Enriched by photoluminescence, magnetic moment, optical absorption, and electron density, metal NPs are quite delicate optical transducers that are able to give much higher absorption cross-sections as well as light scattering compared to usual fluorescing and absorbing dyes, and this makes them suitable for chemical sensing and biosensing [142,143]. Gold NPs usually exhibit strong absorption of light and scattering due to their LSPR (localized surface plasmon resonance) [144]. In photothermal treatment of tumors, it is very appealing to use absorbed light excitation energy conversion [145]. Various magnetic materials like Fe_3_O_4_, MFe_2_O_4_ (M = Mn(II), Zn(II), Ni(II), Co(II), etc.), and even alloys (Fe–Au, Fe–Pt), can be used for the design of these highly tunable magnetic nanostructured materials for application as MRI contrasting agents [146,147,148,149,150]. Furthermore, hyperthermia-based cancer treatment and drug delivery are also supported by using these NPs [151,152,153].

### 4.7. Mesoporous Silica Nanostructured Materials and Their Features

Mesoporous silica nanostructured materials (MSNMs) possess favorable properties for controlled drug release. They are easily synthesized and can be chemically modified on their huge surface area, both on their large surface area and within the pore interiors, which provides high storage capacity for anticancer drugs.

Controlled drug release could be accomplished by “capping”, or better said, by using “nanovalves”, which are organic molecules attached to the pore opening, therefore avoiding release of the material stored, e.g., anticancer drugs. Some organic molecules applied for the nanovalve are rotaxanes and pseudorotaxanes [154]. To cover pore openings, polymers have also been utilized. Another way to provide MSNMs with controlled-release features is to attach active molecules and drugs to the surface of MSNMs by linkages that are stimuli-responsive.

#### 4.7.1. pH Sensitive Activation

Nanoparticles and nanostructured materials pass into cells by endocytosis and are faced with endosomal/lysosomal surroundings with lower pH, and also the tumor interior has lower pH than normal cells owing to hypoxic conditions [139]. This leads to in vitro and in vivo autonomously activated delivery.

One example is that, for instance, DOX drug molecules are attached in MSNMs pores to the inner walls [155], achieving protection. The attachments are actually hydrazone bonds that cannot hold in acidic conditions.

**Figure 4:**
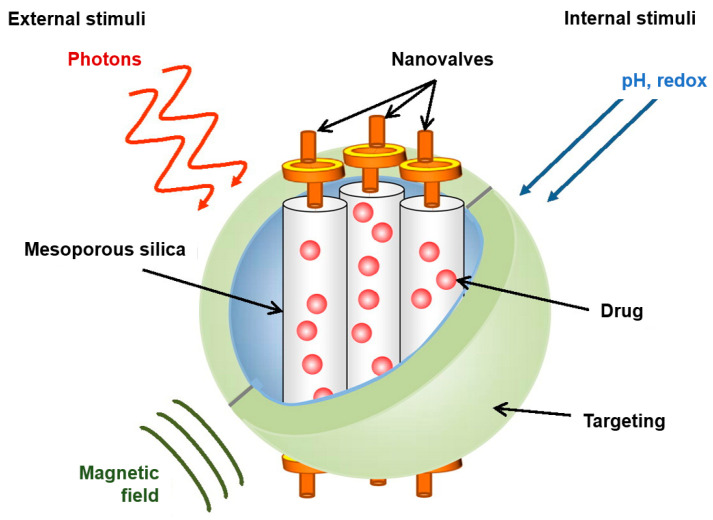
MSNMs with multi-functionality [139].

Different low pH sensitive materials (Figure 4) are used for capping the pore openings: stalk with α-cyclodextrin based on aniline [156], *N*-methylbenzimidazole (MBI) stalk and β-cyclodextrin [157], polyethyleneimine (PEI) as a stalk, and also α-cyclodextrin as a cap [158]. Moreover, an MSNM-based gated unit was obtained by conjugating superparamagnetic Fe_3_O_4_ NPs on the pore exit of MSNs with a reversible linker—a boronate ester linker. Therefore, MSNMs and Fe_3_O_4_ nanoparticles must be functionalized by polyalcohol derivatives and boronic acid. As a result, Fe_3_O_4_ nanoparticles were anchored at pore openings [159]. A low pH makes this capping labile as boroesters hydrolyze.

This gating mechanism to form boronate esters also works for gold NPs. Functionalized AuNPs are then a fine nanoscopic cap [160]. Further, a pH-sensitive nanosystem can be fabricated using the coordination abilities of metal ions [161]. Chitosan [162,163] and pH-responsive lipid compounds are also used for covering pore openings [164].

Modern MSNMs are expected to exhibit both targeting and controlled-release capabilities. Hwang et al. developed MSNMs functionalized with transferrin for targeting and pH-responsive nanovalves, which activate under acidic conditions [156]. Theron et al. [165] created a hydrogen-bonded capping mechanism using complementary nucleic acids. Adenine or uracil was covalently attached to bulky β-cyclodextrin caps. These systems remained stable at neutral pH and released drugs in acidic environments.

Investigations of pH-sensitive polymer nanovalves revealed the appropriate controlled release of loaded molecules [166].

#### 4.7.2. Redox Activated System

Another example of internal stimuli, represented in Figure 4, is the redox condition (in extracellular and cytosolic milieu) [139]. The glutathione concentration varies from 1–6 μM in the bloodstream to 10 mM in the cell, meaning that disulfide bonds will be preserved while in the bloodstream and cleave in the cell. A variety of materials using disulfide bonds were designed and examined. This includes disulfide-containing rotaxane nanovalves [167], and to link nanoparticle caps to openings of the pores [168,169], cyclodextrin [170,171], polymers, and collagen are linked by disulfide bonds [172,173].

#### 4.7.3. Systems That Respond to Light

Drug delivery induced by external light (Figure 4) at a specific location and time could be accomplished with azobenzene [139]. Upon light exposure, azobenzene starts a wagging motion resulting from being between *cis* and *trans* configurations, and this has been applied to control loading–releasing of active drugs through pores [174].

As an alternative to being in pores, azobenzene could be attached to nanovalves [175,176]. However, UV–Vis light is necessary for activation, which is a limitation since desirable wavelengths for tissue exposure are in the spectral range of 800–1100 nm. This can be accomplished by developing TPE (two-photon excitation) that is in the near-infrared region. By avoiding UV–Vis light this way, comparable MSNs using azobenzene in nanovalves, which are also two-photon-responsive, were developed [177,178]. For this same purpose, coumarin derivatives sensitive to light stimuli have been utilized [179,180].

#### 4.7.4. MSNM-Based Drug Release Systems That Respond to Magnetic Field

Magnetic fields can penetrate tissue much better than light, as represented in Figure 4. This possibility can be provided by using an iron oxide nanoparticle [181]. The magnetic core delivers countless new assets to the MSNMs. Exposure to an oscillating magnetic field can heat up the nanostructured materials, and iron oxide has the possibility to boost MRI imaging [139]. Polymers sensitive to temperature change were applied as a shell for iron oxide core MSNMs. Such a polymer was created by functionalization of polyethylene glycol with azo bonds, which break by simply raising the temperature [182]. One more example is polyethyleneimine-bpoly(*N*-isopropylacrylamide), which has a role as a thermosensitive gate guardian [183]. Iron oxide, besides being used as a core, could also find purpose as a cap to prevent drug release, being a magnetic nanoparticle [184].

#### 4.7.5. Multifunctional MSNM Carriers

For the development of multifunctional nanosystems, such as for the delivery of drugs and siRNA (small interfering RNA), MSNs are highly promising. The surface of MSNMs might be coated to transport and distribute siRNA, which may cause cessation of gene expression [185,186]. In a mouse tumor model, MSN carrying siRNA was able to shut down TWIST, one of the hallmarks of the regulators of epithelial–mesenchymal transition [187]. An advanced medical treatment could be developed, for example, if an iron oxide core is present in the center of an MSNM. Such materials are beneficial for MRI enhancement. Nanovalves responsive to the oscillating magnetic field are intended for on-command drug delivery from inside mesoporous structures. Moreover, using polyetherimide (PEI) for coating of MSNMs and targeting moiety, siRNA was delivered to target cells. In this way, MSNMs should enable futuristic theranostics, which is expected to accomplish by nanoscience researchers.

Nanostructured mesoporous materials, for instance MCM-41 and SBA-15 (Mobil Composition of Matter No. 41 and Santa Barbara Amorphous 15, respectively), have been the subjects of studies in various biological applications. They can be loaded with many compounds [188]. The natural product emodin (1,3,8-trihydroxy-6-methylanthraquinone, abbreviated as EO) exhibits anti-inflammatory, antineoplastic, and antiangiogenesis properties both in vitro and in vivo. The fluorescence of EO, as well as its biological properties, makes it an attractive candidate for pharmacological as well as pharmacokinetic studies. Recently, in order to intensify selectivity to cancer cells, immobilization of EO into nonfunctionalized and fluorescent-labeled (with *N*-methylisatoic anhydride or lissamine rhodamine B sulfonyl) spherical mesoporous nanoparticles/nanomaterials (SNMs) was reported. As expected for MSNMs, SNM against the human colon HT-29 cell line was found inactive. SNM carrying EO exhibited comparable activity to EO alone. SNM enters the tumor cells within 2 h, followed by drug release in 48 h. EO immobilized into SNM induces apoptosis in HT-29 cells [189].

Five SBA-15 nanostructured materials were prepared to contain different amounts of EO. 8–36%) [190]. SBA-15 hinders the photodegradation of EO and impedes its release from the vehicle in an extremely acidic milieu. MSNMs containing up to 27% of EO (SBA-15|EO3) showed a very good correlation between the amount of EO in SBA-15 and the viability of melanoma tumor cells (A375, B16, and B16F10) upon treatment. Applying MSNM with a higher grafting rate of EO (32 or 36%) did not significantly change the viability of melanoma cells in comparison to material containing 27% of EO. The mechanism of action against melanoma cells involves caspase-triggered apoptosis, inhibition of Bim and Bcl-2, along with overexpression of Bax and PARP (poly-(ADP-ribose)-polymerase cleavage fragment. Moreover, the same mesoporous silica in the brown tail moth *Euproctis chrysorrhoea* (L.), larvae of a polyphagous insect pest, upregulated glutathione reductase and *S*-transferase, as well as catalase and superoxide dismutase, and thus antioxidative enzymes [191].

## 5. Nanodrugs in Clinical Trials: Polymeric and Lipid Nanocarriers of Platinum Drugs

Improvement of the drug delivery method for all platinum agents is mandatory due to the well-known side effects and drug resistance. The innovative research on the delivery of anticancer drugs or genes using carriers such as polymer [34,192,193,194] and lipids brought scientists closer to an ideal platinum drug carrier, which should be able to load the drug efficiently with zero premature leakage and release the anticancer cargo to its targeting spot in a controlled aspect [192,195]. As a result, various platinum drug delivery matrices have been developed.

Concerning polymeric carriers, it is important to mention the development of AP5346–polymeric oxaliplatin, which is water-soluble and has 17% drug loading with a 23 h blood circulation half-life, and is now in clinical trial phase II [196,197,198]. ProLindac™ (AP5346) is a polymeric oxaliplatin prodrug conjugated to hydroxypropylmethacrylamide (HPMA), a 25 kDa polymer. A pH-sensitive linker, sensitive to a low pH milieu as found usually in most tumors, releases faster platinum species. It has completed phase I trials in patients with solid tumors and a phase I/II study in patients with recurrent ovarian cancer, showing promising efficacy and safety. Future plans include the combinatory use with paclitaxel [138]. NC-6004 is a cisplatin-loaded polymeric micelle (diameter 28 nm, 39% drug loading) currently in phase II and III clinical trials. It has a blood circulation half-life of 10 h [123,199,200,201].

The development of lipid nanocarriers for efficient drug delivery is also a growing field in research that improves anticancer agents [202]. Another liposomal formulation, with a particle size of 110 nm, carrying cisplatin (8.9%), has a blood circulation half-life of 7 h [203]. Promising results have been obtained against non-small cell lung cancer adenocarcinomas when combined with paclitaxel, with even superior activity than that of cisplatin. The drug successfully passed human clinical trials in phases I–III [204].

## 6. Platinum Drugs Immobilized into Inorganic Particles

### 6.1. Gold Nanoparticles

Gold nanoparticles, unique in size, shape, and optical and electronic properties, are presented as potential carriers for delivery for both therapeutic and diagnostic purposes [205]. The work presented by Wheate et al. describes chelated platinum(II) species, oxaliplatin active components, coordinated through thiolated polyethylene glycols(PEG) monolayer [206]. These gold nanoparticles showed an improved cell dismantling effect in comparison to oxaliplatin alone against HT29, HCT116, HCT15, and RKO cell lines. The same research group used gold nanoparticles to deliver cisplatin with improved effects [207,208]. Cisplatin(IV) prodrug, reported by Lippard et al., is immobilized through a thiolated oligonucleotide linker to gold nanoparticles [209]. Cisplatin showed an IC_50_ concentration of 11 µM against the A549 cell line, while the Pt-DNA-Au nanosystems were found to be at least 10 times more active (IC_50_ = 0.9 μM). Platinum(IV) prodrugs were also tethered to gold nanorods [210], which resulted in boosted platinum uptake in the treatment of lung cancers. Those nanostructured materials were able to overcome cisplatin resistance and caused a reduction in glutathione-mediated detoxification.

### 6.2. Fe_3_O_4_ Nanoparticles

Nanoparticles based on iron oxide have been extensively applied as delivery vehicles for platinum drugs. Fe_3_O_4_, a biocompatible magnetite nanoparticle, was predominantly used due to its special magnetic resonance characteristics and field-mediated targeting for therapeutic and diagnostic purposes [211,212]. Sun et al. described hollow iron oxide nanoparticles with immobilized cisplatin. The drug release kinetics can be controlled by tuning of medium pH and the pore sizes of the cavity. Herceptin-coupled hollow iron oxide nanoparticles loaded with cisplatin exhibited higher activity against breast cancer SK-BR-3 cells (IC_50_ = 2.9 μM) than cisplatin (IC_50_ = 6.8 μM) needed for free cisplatin [213,214]. Moreover, the same NPs were able to target SK-BR-3 cells. Superparamagnetic iron oxide nanoparticles functionalized with carboxy-methylcellulose, possessing COO^–^ function, were coordinated to cisplatin and used for drug delivery [215]. Iron oxide NPs, encapsulated in gelatin, were used as a vehicle system for platinum(IV) prodrug delivery. Theranostic potential for these NPs has been reported, showing capability for MRI imaging as well as enzyme-stimulated drug release [216].

### 6.3. Mesoporous Silica Nanostructured Materials

**Figure 5:**
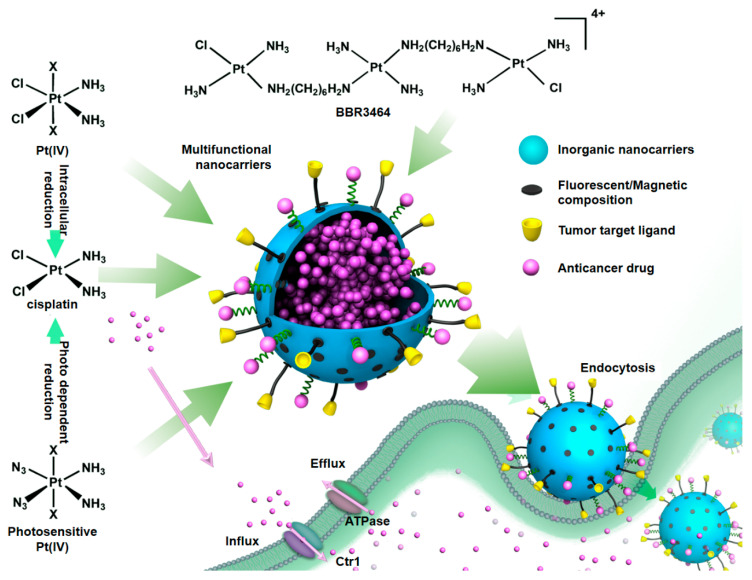
An example of possible multifunctional inorganic nanoparticles for platinum drug delivery. Fluorescent imaging agents, tumor targeting ligands, and anticancer platinum drugs such as platinum(II), platinum(IV), and multinuclear platinum drugs can be used for drug delivery and imaging. Platinum(IV) prodrugs can be reduced to cytotoxic platinum(II) by intracellular and extracellular stimuli [138].

Due to their large surface area per mass unit, porous mesostructure, tunable pore size, easy surface modification, biocompatibility, and so much more, mesoporous silica nanostructured materials (MSNMs) have been a subject of extensive research [217,218]. MSNMs are already used in medicine for the delivery of anticancer drugs, including doxorubicin [219], paclitaxel [220], and cisplatin [221]. A high density of COOH groups was introduced by Huang et al. into the inner surface of MSNMs. Afterwards, COO^–^ was used to coordinate active oxaliplatin species [222]. A pH-sensitive coordination bond, COO–Pt at pH 5 triggered the release of oxaliplatin species. Immobilized oxaliplatin exhibited better cytotoxicity against HepG-2 cancer cells than oxaliplatin alone, presumably because of the higher platinum intracellular uptake, which might cause Pt-DNA adduct formation to a higher extent, and eventually, failure of DNA repair might occur. On the other hand, transplatin is not active against solid tumors due to the side reactions in the bloodstream, interactions with biomolecules containing thiol groups, as well as unstable Pt-DNA adducts. The work of Tao et al. describes the immobilization of cisplatin and transplatin into MCM-41 and SBA-15 MSNs. Surprisingly, the similar activity of both the transplatin and cisplatin-loaded MSNMs was observed, which is another proof of the advantages of nano-scale drug vehicles (Figure 5) [223,224].

Moreover, other researchers successfully loaded cisplatin into SBA-15 MSNMs with different grafting rates, thus showing favorable results [225,226]. Free and immobilized cisplatin was evaluated in vitro and in vivo against B16F1 low metastatic mouse melanoma. As reported previously, the drug vehicle SBA-15 was inactive. On the other hand, free and loaded cisplatin, similar in antitumor activity, exhibited high cytotoxicity in the lower μM range. The mechanism of action for cisplatin and appropriate MSNMs inhibited the cell proliferation of B16F1 cells, arresting them in the G2/M phase of the cell cycle.

Apoptosis mediated by caspase activation and NO (nitric oxide) elevation was determined as the main mode of cell death. To some extent, elevated autophagy could also be detected, while ROS production (reactive oxygen species) was not affected upon the treatment of B16F1 cells. Survived clones transit into senescent cells. In vivo studies in C57BL/6 mice showed greater potential of cisplatin immobilized into SBA-15, whereas naked cisplatin did not hinder melanoma growth. Furthermore, nephron- and hepatotoxicity in animals treated with cisplatin were confirmed, contrary to SBA-15 loaded with cisplatin, where those toxicities were reduced. Thus, pointing out improved antitumor potential of cisplatin when immobilized into SBA-15 [225].

The same drug-vehicle system was evaluated in a panel of four tumor cell lines: human prostate (PC3), colon (HT-29), and adenocarcinoma (HeLa), as well as mouse malignant melanoma (B16F10). Additionally, MSNMs carrying cisplatin (ca. 3.5 times lower IC_50_ concentration) were found to exhibit superior inhibition along with senescence induction in B16F10 cells than cisplatin. On the other hand, SBA-15 loaded with inactive K_2_[PtCl_4_] complex did not show anticancer activity. Induction of senescence may be a harmless method in cancer therapy, while senescent cells can be removed by immune cells. Up to now, drugs inducing apoptosis are in use in clinical treatments. Understanding the senescence induced by antitumor drugs will permit oncologists to discover this tumor suppressor mechanism [226].

Platinum(IV) conjugates having cisplatin scaffold and in axial positions, ibuprofen, flurbiprofen, or naproxen (nonsteroidal anti-inflammatory drugs—NSAIDs) [227] as well as derivatives of caffeic and ferulic acids [228] were recently prepared and immobilized into SBA-15. Conjugates as well as appropriate MSNMs exhibited superior antiproliferative activity (up to 1.000 fold) against breast MCF-7, MDA-MB-468, BT-474, and HCC1937 cancer cell lines in comparison to cisplatin. Furthermore, in contrast to cisplatin, conjugates and MSNMs containing NSAIDs showed higher potential against MDA-MB-468 [227]. In addition, apoptosis, elevated autophagy, and upregulation of NO and ROS were found for conjugates containing naproxen.

The most potent conjugate cisplatin-*bis*(diacetyl caffeate) and appropriate MSNMs in SBA-15 were active against mouse breast cancer cell line 4T1 and showed that these compounds induce apoptotic cell death, causing strong caspase activation. In vivo, in BALB/c mice, 1 and SBA-15|1 inhibited tumor growth while decreasing the necrotic area and lowering the mitotic rate.

The most potent free and immobilized conjugates into SBA-15 cisplatin-*bis*(diacetyl caffeato) were also active against mouse breast cancer cell line 4T1, inducing apoptosis by activating caspases [228]. Furthermore, an appropriate in vivo model (BALB/c mice) reveals that both free and loaded cisplatin conjugate inhibited tumor growth in experimental animals along with decreased necrotic area and the mitotic rate.

## 7. Mesoporous Silica Nanostructured Materials as Carriers of Non-Platinum Metal-Based Complexes

### 7.1. Nanostructured Materials Functionalized with Palladium Complexes

Using gentle stirring and various [PdCl_2_(cod)] (cod = 1,5-cyclooctadiene) amounts, loading of palladium(II) complex was performed in MSNMs (SBA-15 and MSU-2), yielding MSNMs containing palladium hybrids (SBA-15–Pd and MSU-2–Pd). SBA-15–Pd and MSU-2–Pd were evaluated for their catalytic potential in Suzuki–Miyaura *C*–*C* coupling reactions between 2-bromopyridine with 4-carboxyphenylboronic acid and 3-bromoanisole with 4-carboxyphenylboronic acid. Moderate conversion rates were observed with MSNMs containing palladium hybrids. These materials displayed a good level of recyclability, catalyzing in five successive catalytic analyses. Moreover, the antiproliferative activity potential of SBA-15–Pd and MSU-2–Pd hybrids against head and neck cancer A253, ovarian cancer A2780, anaplastic thyroid cancer 8505C, colon cancer DLD-1, and lung carcinoma A549 cell lines has been assessed, pointing to high antiproliferative activities [229].

### 7.2. Nanostructured Materials Functionalized with Ruthenium Complexes

SBA-15 and SBA-15~SH (functionalized SBA-15 with 3-mercaptopropyltriethoxysilane) mesoporous silica nanostructured materials were loaded with [Ru(η^6^-*p*-cymene)Cl_2_{Ph_2_P(CH_2_)_3_SPh-κ*P*}] complex, abbreviated as [Ru]. Neither carrier of MSNMs exhibited cytotoxic activity against mouse melanoma cells with low B16 and high B16F10 metastatic potential. In contrast, [Ru] complex immobilized in MSNMs showed extraordinary inhibition potential against both mouse melanoma cell lines. MSNMs with [Ru] exhibited 3–6 times higher Ru content activity than free [Ru], thus having IC_50_ concentrations between 1 and 2 μM. MSNMs modulated different apoptotic modes of cell death in comparison to [Ru] by inducing caspase activation, which is unlikely to [Ru]. This points out the influence of functionalization on the mechanism of action. However, one of the similarities between naked and immobilized drugs is the suppression of autophagy in B16 cells [230].

Through a pH-responsive hydrazone linkage on MSNMs, 2-thienylmethyl)hydrazine or (5,6-dimethylthieno[2,3-d]pyrimidin-4-yl)hydrazine were covalently bonded to functionalized MSNMs [231]. Monomers, [Ru], of dichlorido(*p*-cymene)ruthenium(II) dimmer were coordinated to the MSNMs. Enhanced release of [Ru] species occurs to a higher extent in a medium with a lower pH. In contribution to that, MSNMs soaked in weak acidic conditions showed higher killing potential on B16F1 melanoma cells.

A series of MSNMs have been prepared and loaded with photoactive ruthenium(II) complex bearing 2,2′-bipyridine (bipy) and dipyrido[3,2-*a*:2′,3′-*c*]phenazine (dppz) ligands, [Ru(bipy)_2_-dppz-7-hydroxymethyl][PF_6_]_2_ using different methods [232]. Depending on the synthetic procedure used in the immobilization process of [Ru(bipy)_2_-dppz-7-hydroxymethyl][PF_6_]_2_ into MSNMs, the incorporation occurred in a diverse way. In addition, this caused changes in the textural and structural parameters of MSNMs. As a model, HeLa cervical cancer cells were used to examine the phototherapeutic potential of MSNMs containing [Ru(bipy)_2_-dppz-7-hydroxymethyl][PF_6_]_2_. Promising results for ruthenium-functionalized materials were observed [232].

### 7.3. Nanostructured Materials Functionalized with Titanium Complexes

In one of the prominent pioneer studies, MCM-41 and SBA-15 were loaded with two different titanocene complexes. The cytotoxicity of MSNMs containing titanium(IV) complexes was evaluated using K562, HeLa, Fem-x, as well as PBMCs (normal immunocompetent cells) [233]. Carrier MSNMs, MCM-41 and SBA-15, did not show activity against the investigated cell lines. However, the high in vitro potential of two classes of titanium complexes—budotitane and titanocene dichloride—showed anticancer activity against a broad spectrum of cancer cells (leukemia L1210 and P388, lung carcinoma LLCs, colon adenocarcinoma MC38, and others) and in several in vivo mouse models [234].

The immobilization of two titanocene complexes into SBA-15, functionalized with 1-methyl-3-(triethoxysilylpropyl)imidazolium chloride (ionic liquid), was recently reported [235]. Cyclic voltammetry was employed to determine the impact of the ionic liquid on the reduction potential of titanium(IV) complexes. Additionally, the interaction with transport/serum proteins (e.g., transferrin, BSA) and with target molecules (e.g., single- and double-stranded DNA, guanosine) as well as stability studies in physiological media of MSNMs loaded with titanocene compounds were explored. Biomolecules present in the medium altered the reduction potential value of Ti(IV)/Ti(III) from MSNMs. Moreover, these MSNMs displayed a lower affinity for guanosine or DNA than for transport/serum proteins [235].

Aminodiol 3-[bis(2-hydroxyethyl)amino]propyltriethoxysilane was grafted onto SBA-15 (→SBA-PADOH) [236]. Two titanium-based complexes, [Ti(η^5^-C_5_H_5_)_2_Cl_2_], ([Ti(η^5^-C_5_H_5_)(η^5^-C_5_H_4_CHPhNf)Cl_2_] (Nf = C_10_H_7_), as well as one tin(IV) compound, SnPh_2_Cl_2_, were immobilized into SBA-PADOH. All MSNMs have been characterized by various traditional techniques for solid-state chemistry. In vitro studies were conducted using A431 epidermoid carcinoma, A2780 ovarian carcinoma, and human DLD-1 colon carcinoma. Depending on functionalization and the used metal complexes, the apoptotic pathway was triggered in a different manner. Thus, MSNMs containing titanocene derivatives triggered apoptosis by interfering with the TNF-α (tumour necrosis factor alfa) pathway, while material with organotin(IV) compound through the Fas-FasL (tumour necrosis factor receptor 6) system [236].

### 7.4. Nanostructured Materials Functionalized with Tin Complexes

In recent years, MSNMs containing organotin(IV) compounds have been evaluated as potential drug delivery systems in the fight against cancer. Almost ten years ago, it was shown that organotin(IV) compounds immobilized into MSNMs can be applied as a biocompatible strategy for cancer treatment [237]. Kaluđerović et al. loaded Ph_3_Sn(CH_2_)_6_OH into SBA-15 chemically modified with (3-chloropropyl)triethoxysilane. Such MSNMs were able in vivo (syngeneic C57BL/6 mice) to totally diminish the tumor in experimental animals. Contrary to apoptosis, this presents safe eradication of tumors without undesirable compensatory proliferation, which may occur the apoptosis is triggered. Furthermore, organotin(IV)-MSNMs distinguished between normal and tumor cells, thus making them non-toxic towards normal tissues. In vitro studies on mouse melanoma B16 cells pointed out that the main mechanism of action is JNK-independent apoptosis, while surviving cells evolved into a melanocyte-like non-proliferative phenotype [225,237,238,239,240].

The same group investigated how functionalization of SBA-15 (3-chloropropyl)triethoxysilane, SBA-15 → SBA-15~Cl and (3-aminopropyl)triethoxysilane, SBA → SBA-15~NH_2_) with physically adsorbed Ph_3_Sn(CH_2_)_6_OH affects the mechanism of action on B16 cells [238]. Moreover, fluorescent MSNMs (bearing isatoic moiety, → SBA-15~NF) loaded Ph_3_Sn(CH_2_)_6_OH were prepared for cellular uptake study. After only 2 h, fluorescent MSNMs carrying organotin(IV) compound entered the B16 cells and were allocated in the cytoplasm. MSNMs without organotin(IV) compound (SBA-15, SBA-15~Cl, SBA-15~NH_2_) were inactive against B16 cells. Functionalization of SBA-15 did not affect the efficacy of the tested drug. However, functionalization impacted the triggered mechanism in melanoma B16 cells. Nonmodified SBA-15-loaded Ph_3_Sn(CH_2_)_6_OH induced massive apoptosis. B16 cells treated with SBA-15~NH_2_|Ph_3_Sn(CH_2_)_6_OH, on the one hand, exhibited lower caspase activity and obstructed apoptosis in time, and on the other, triggered autophagy (process opposed to apoptosis). Notably, only SBA-15~Cl carrying Ph_3_Sn(CH_2_)_6_OH was able to differentiate B16 melanoma cells into melanocytes [238].

Cellular uptake, mechanism of action, and influence on the stemness of human melanoma cell line A375 were investigated using Ph_3_Sn(CH_2_)_6_OH immobilized into [239]. Internalization of cylindric MSNMs of relatively large size occurred with stable adhesion only after 15 min. Two main uptake mechanisms of MSNMs into A372 cells within 2 h were identified: macropinocytosis and passive fluid-phase uptake. Organotin(IV) loaded SBA-15~Cl induced caspase-dependent apoptosis along with senescence in A375 cells. Upon treatment, Schwann-like phenotype in the subpopulation of cells could be identified, whereas Wnt, Notch1, and Oct3/4 were regulated in the direction of less aggressive signature, thus affecting the signaling pathway in control for safeguarding of invasiveness and pluripotency. Also here, MSNMs enhanced the efficiency of organotin(IV) compound by effective uptake and intracellular response, as well as declined stem characteristics of extremely invasive human melanoma A375 cells [239].

An analogous Ph_3_Sn(CH_2_)_6_OH compound with a shorter CH_2_ spacer between Ph_3_Sn moiety and OH function, namely, Ph_3_Sn(CH_2_)_3_OH, was loaded into SBA-15~Cl and in vitro potential of free and immobilized organotin(IV) compound was tested against the A2780 high-grade serous ovarian carcinoma cell line [240]. Internalization of both MSNMs as well as organotin(IV) compound internalization occurs in the A2780 cells with a comparable mechanism of action. Moreover, Ph_3_Sn(CH_2_)_3_OH and appropriate MSNMs induced A2780 cell activation of caspases 2, 3, 8, and 9 and apoptosis and accumulation of the cells in the sub-G1 phase of the cell cycle and enhanced ROS production. However, immobilized organotin(IV) compound in MSNMs with 3.5 times lower concentration exhibited the same effect as Ph_3_Sn(CH_2_)_3_OH on A2780 cells, indicating that SBA-15~Cl boosted the activity of the drug. Conversely, only SBA-15~Cl|Ph_3_Sn(CH_2_)_3_OH strongly disturbed the clonogenic potential and mobility of A2780, which most probably suppresses p-38 MAPK. On the other hand, a much weaker impact of Ph_3_Sn(CH_2_)_3_OH could be caused by dissimilar alteration of STAT-3 and p-38 expression [240].

In 2022, there was a study on the influence of the chain length in free and immobilized Ph_3_Sn(CH_2_)*_n_*OH (*n* = 3, 4, 6, 8 and 11) in SBA-15~Cl on the cytotoxic activity of (alkyl-ω-ol)triphenyltin(IV) on the in vitro activity against mouse melanoma B16, human melanoma A375, and mouse colon cancer CT26CL25 cell lines [240]. In all cases, a higher in vitro potential was observed when (alkyl-ω-ol)triphenyltin(IV) immobilized into MSNMs was used, with the highest activity of SBA-15~Cl|Ph_3_Sn(CH_2_)_8_OH. Furthermore, besides apoptosis, these MSNMs were able to induce the differentiation of B16 melanoma cells into less aggressive and more mature phenotypes by upregulating ROS and/or RNS production. In vivo experiments in C57BL6 mice (syngeneic mouse model of melanoma) pointed out that immobilization of Ph_3_Sn(CH_2_)_8_OH was beneficial since a decrease in tumor volume was detected upon treatment with MSNMs containing the tetraorganotin(IV) compound.

Several theranostic MSNMs containing two active agents, an organotin compound and chlorambucil, as well as the fluorescent marker Alexa Fluor 647, were prepared. From the in vitro investigations of the two drugs on triple-negative breast cancer MDA-MB-231 cells, a synergistic effect and higher cell migration inhibition could be observed. In vivo experiments identified selective accumulation of MSNMs in the tumor. In addition, the beneficial effect on the nanotoxicity of the combinatory therapy (organotin compound and chlorambucil) when applied to immobilized MSNMs in vivo was found [241].

A recently published article from Gómez-Ruiz et al. describes the synthesis of MSNMs, functionalization with (3-mercapto)triethoxysilane, and immobilization in Ph_3_SnCl, as well as biological activity against tumors MCF-7, SKOV3, and B16F10 and normal HEK and CHO cells. MSNMs loaded with Ph_3_SnCl were integrated by pinocytosis or endocytosis in MCF-7 cells, triggering ROS formation and apoptosis, which was not the case in the treatment of normal CHO cells. In the chick embryo model, these MSNMs revealed anti-angiogenic properties. The genotoxicity investigation of MSNMs pointed out the aneugenic and clastogenic effects predominantly in CHO cells treated with high concentrations [242].

Gómez-Ruiz et al. reported on organotin(IV) and titanium(IV) compounds loaded into MSNMs, which were functionalized with transferrin (targeting molecule) and fluorescein isothiocyanate (image agent). Mesoporous silica nanostructured materials loaded with organotin(IV) compounds, some of them even 50 times more active than the clinically used drug oxaliplatin, were superior to materials loaded with titanium(IV) complexes. Uptake studies reveal elevated uptake of MSNMs containing titanium(IV) complexes in comparison to those carrying organotin(IV) compounds, which might be explained by stronger interaction with transferrin and therefore more efficient internalization of MSNMs in the cells. Finally, the study showed that organotin(IV) loaded into MSNMs was able to regulate nuclear factor κβ transcription factor (NF-κβ), human fibroblast growth factor 2 (FGF-2), and vascular endothelial growth factor A (VEGF-A) in A2780 cells, revealing anti-angiogenic effects through FGF-2 and VEGF-A, in addition to the interaction of transferrin with MSNs [243].

In summary, metal-based complexes immobilized or functionalized into/onto nanomaterials represent a powerful new generation of drug carriers capable of navigating all critical phases of delivery. This includes drug loading, systemic circulation, targeted release, and cellular response. Their multifunctional design paves the way for safer, smarter, and more effective cancer therapies.

## 8. Conclusions

Despite the lack of standardized synthesis strategies for designing novel active metallodrugs, the urgent need for more effective and efficient therapeutic and diagnostic tools in healthcare remains a strong driving force.

The design of powerful new drugs for chemotherapy treatment of cancer is a major priority in different research areas that include natural products, biochemistry, molecular biology, medicine, pharmacology, and chemistry. This review has provided an overview of nanoparticles and nanostructured materials from simple to very complex, as promising tools for achieving targeted delivery of active agents. Particular attention was given to the functionalization of so-called “magic bullets” and their role in improving cancer therapy. Advances in nanocarrier research have led to the emergence of novel nanodrugs now progressing through clinical trials. Nanoscale drug delivery is nowadays a predominantly explored field of research. The earliest nanoscale systems to be advanced for drug delivery were liposomes; thus, the FDA and general clinical approval of liposomal formulations of doxorubicin and vincristine confirm that efforts in this area of science are fruitful. Lipoplatin is known as the liposomal formulation of cisplatin, and good progression in clinical trials indicates that it is becoming the next great platinum-based drug. We can predict a renewed growing excitement in exploring platinum agents and, above all, nanoparticle formulations.

Several nanosystems show more control in the release of the loaded drugs, being subjected to external and internal stimuli—pH variations, temperature, magnetic field, light, etc. The activity and selectivity are effectively enhanced when nanodrugs are conjugated with particular ligands on their surfaces, known as targeting overexpressed receptors.

From a structure–activity relationship (SAR) perspective, minor structural changes to the drug, such as the introduction of a methylene (CH_2_) group or grafting modifications on the carrier, can significantly influence encapsulation efficiency, drug–carrier interactions, and release kinetics. Moreover, these modifications not only affect pharmacodynamics but also play a key role in drug delivery system–cell interactions. Remarkably, the same carrier system may induce apoptosis, autophagy, tumor cell differentiation, or even combined responses depending on its surface functionalization. Therefore, careful consideration of SAR principles during the design of nanomaterial-based drug delivery systems is essential for optimizing therapeutic outcomes and achieving selective biological responses.

The problematic issue is still the improvement of drug selectivity. Collective efforts in research by clinicians, chemists, biologists, and pharmacists should upgrade the level of new NP formulations with multiple targeting biomolecules attached and further synergistic forms of metal and non-metal drugs. It is possible that this example could provide precise loading, transporting, and releasing, and ultimately improve tumor cytotoxicity with reduced toxicity to healthy cells. Wide applications of multifunctional nanostructures should not be limited to the delivery of metallodrugs. It is of high importance to develop an all-in-one type of drug soon, possessing controlled delivery, targeting, imaging, etc. These nanosystems can, in due course, make it possible to detect and destroy cancer cells in a non-toxic way, as desired for a long time.

The metal complexes have an advantage in adaptable ligand substitution reactions, chosen ligand and coordination numbers, a variety of redox states, ligand exchange induced by light, emitting isotopes, and so on. It is no secret that we still have a long way to go to achieve the final goal, but encouraging results were attained recently, so we know the direction is right. The greatest proof of efficiency is combining nanostructured materials, metal complexes, and biomolecules.

Regrettably, unsolved problems arise with the safety of nanostructured materials themselves, having no or very little adequate knowledge about the topic. Without doubt, it is an obligation to carry out detailed research of the pharmacokinetic profiles of a huge number of nanoparticles and nanomaterials, and creating databases of health hazards and all other necessary information would be very valuable.

Finally, computational tools such as molecular dynamics, docking simulations, and predictive pharmacokinetic modeling have emerged as powerful allies. These in silico approaches accelerate development, reduce experimental workload, and help predict therapeutic outcomes, bridging the gap between discovery and clinical application.

## Data Availability

Not applicable.

## References

[1] Sung H., Ferlay J., Siegel R.L., Laversanne M., Soerjomataram I., Jemal A., Bray F. (2021). Global Cancer Statistics 2020: GLOBOCAN Estimates of Incidence and Mortality Worldwide for 36 Cancers in 185 Countries. CA Cancer J. Clin..

[2] American Cancer Society (2024). Global Cancer Facts & Figures.

[3] Ferlay J., Soerjomataram I., Dikshit R., Eser S., Mathers C., Rebelo M., Parkin D.M., Forman D., Bray F. (2015). Cancer Incidence and Mortality Worldwide: Sources, Methods and Major Patterns in GLOBOCAN 2012. Int. J. Cancer.

[4] Vogelstein B., Kinzler K.W. (2004). Cancer Genes and the Pathways They Control. Nat. Med..

[5] Rodríguez F., Caruana P., De la Fuente N., Español P., Gámez M., Balart J., Llurba E., Rovira R., Ruiz R., Martín-Lorente C. (2022). Nano-Based Approved Pharmaceuticals for Cancer Treatment: Present and Future Challenges. Biomolecules.

[6] Hartmann J.T., Lipp H.-P. (2003). Toxicity of Platinum Compounds. Expert Opin. Pharmacother..

[7] Kosmider B., Wyszynska K., Janik-Spiechowicz E., Osiecka R., Zyner E., Ochocki J., Ciesielska E., Wasowicz W. (2004). Evaluation of the Genotoxicity of Cis-Bis(3-Aminoflavone)Dichloroplatinum(II) in Comparison with Cis-DDP. Mutat. Res./Genet. Toxicol. Environ. Mutagen..

[8] Perán M., García M.A., López-Ruiz E., Bustamante M., Jiménez G., Madeddu R., Marchal J.A. (2012). Functionalized Nanostructures with Application in Regenerative Medicine. Int. J. Mol. Sci..

[9] Goldberg M., Langer R., Jia X. (2007). Nanostructured Materials for Applications in Drug Delivery and Tissue Engineering. J. Biomater. Sci. Polym. Ed..

[10] Wang X., Yang L., Chen Z., Shin D.M. (2008). Application of Nanotechnology in Cancer Therapy and Imaging. CA Cancer J. Clin..

[11] Patel S.P., Patel P.B., Parekh B.B. (2014). Application of Nanotechnology in Cancers Prevention, Early Detection and Treatment. J. Cancer Res. Ther..

[12] Sun T., Zhang Y.S., Pang B., Hyun D.C., Yang M., Xia Y. (2014). Engineered Nanoparticles for Drug Delivery in Cancer Therapy. Angew. Chem. Int. Ed..

[13] Lu J., Liong M., Zink J.I., Tamanoi F. (2007). Mesoporous Silica Nanoparticles as a Delivery System for Hydrophobic Anticancer Drugs. Small.

[14] Mitchell M.J., Billingsley M.M., Haley R.M., Wechsler M.E., Peppas N.A., Langer R. (2021). Engineering Precision Nanoparticles for Drug Delivery. Nat. Rev. Drug Discov..

[15] Carissimi G., Montalbán M.G., Fuster M.G., Víllora G., Pham P.V. (2021). Nanoparticles as Drug Delivery Systems. 21st Century Nanostructured Materials.

[16] Lombardo D., Kiselev M.A., Caccamo M.T. (2019). Smart Nanoparticles for Drug Delivery Application: Development of Versatile Nanocarrier Platforms in Biotechnology and Nanomedicine. J. Nanomater..

[17] Zeng Z., Gao H., Chen C., Xiao L., Zhang K. (2022). Bioresponsive Nanomaterials: Recent Advances in Cancer Multimodal Imaging and Imaging-Guided Therapy. Front. Chem..

[18] Jadhav V., Roy A., Kaur K., Rai A.K., Rustagi S. (2024). Recent Advances in Nanomaterial-Based Drug Delivery Systems. Nano-Struct. Nano-Objects.

[19] Kuskov A.N., Kukovyakina E.V. (2025). Nanotechnology-Based Drug Delivery Systems, 2nd Edition. Pharmaceutics.

[20] Estanqueiro M., Amaral M.H., Conceição J., Sousa Lobo J.M. (2015). Nanotechnological Carriers for Cancer Chemotherapy: The State of the Art. Colloids Surf. B Biointerfaces.

[21] Poon W., Zhang X., Nadeau J. (2014). Nanoparticle Drug Formulations for Cancer Diagnosis and Treatment. Crit. Rev. Oncog..

[22] Puttasiddaiah R., Basavegowda N., Lakshmanagowda N.K., Raghavendra V.B., Sagar N., Sridhar K., Dikkala P.K., Bhaswant M., Baek K.-H., Sharma M. (2025). Emerging Nanoparticle-Based Diagnostics and Therapeutics for Cancer: Innovations and Challenges. Pharmaceutics.

[23] Saleh T.A. (2020). Nanomaterials: Classification, Properties, and Environmental Toxicities. Environ. Technol. Innov..

[24] Seeta Rama Raju G., Benton L., Pavitra E., Yu J.S. (2015). Multifunctional Nanoparticles: Recent Progress in Cancer Therapeutics. Chem. Commun..

[25] Trucillo P. (2022). Drug Carriers: A Review on the Most Used Mathematical Models for Drug Release. Processes.

[26] Kolmakov A., Moskovits M. (2004). Chemical Sensing and Catalysis by One-Dimensional Metal-Oxide Nanostructures. Annu. Rev. Mater. Res..

[27] Al-Douri Y., Al-Douri Y. (2022). Electrical and Optical Properties of Nanomaterials. Nanomaterials: Basics to Applications.

[28] Kumar A., Shahvej S., Yadav P., Modi U., Yadav A.K., Solanki R., Bhatia D. (2025). Clinical Applications of Targeted Nanomaterials. Pharmaceutics.

[29] Qiu L.Y., Bae Y.H. (2006). Polymer Architecture and Drug Delivery. Pharm. Res..

[30] Svenson S., Tomalia D.A. (2005). Dendrimers in Biomedical Applications--Reflections on the Field. Adv. Drug Deliv. Rev..

[31] Langer R. (1998). Drug Delivery and Targeting. Nature.

[32] Al-Zoubi M.S., Al-Zoubi R.M. (2022). Nanomedicine Tactics in Cancer Treatment: Challenge and Hope. Crit. Rev. Oncol./Hematol..

[33] Rahman M.A., Jalouli M., Yadab M.K., Al-Zharani M. (2025). Progress in Drug Delivery Systems Based on Nanoparticles for Improved Glioblastoma Therapy: Addressing Challenges and Investigating Opportunities. Cancers.

[34] Duncan R. (2003). The Dawning Era of Polymer Therapeutics. Nat. Rev. Drug Discov..

[35] Rehn S.M., Gerrard-Anderson T.M., Chen Y., Wang P., Robertson T., Senftle T.P., Jones M.R. (2023). Surface Ligands Dictate the Mechanical Properties of Inorganic Nanomaterials. ACS Nano.

[36] Al-Thani A.N., Jan A.G., Abbas M., Geetha M., Sadasivuni K.K. (2024). Nanoparticles in Cancer Theragnostic and Drug Delivery: A Comprehensive Review. Life Sci..

[37] Sahoo S.K., Labhasetwar V. (2003). Nanotech Approaches to Drug Delivery and Imaging. Drug Discov. Today.

[38] Vasir J.K., Reddy M.K., Labhasetwar V.D. (2005). Nanosystems in Drug Targeting: Opportunities and Challenges. Curr. Nanosci..

[39] Kipp J.E. (2004). The Role of Solid Nanoparticle Technology in the Parenteral Delivery of Poorly Water-Soluble Drugs. Int. J. Pharm..

[40] Rabinow B.E. (2004). Nanosuspensions in Drug Delivery. Nat. Rev. Drug Discov..

[41] Hassan T., Huang X., Zhou C., Sikander M., Khan M., Saeed S. (2022). Nanoparticles in Cancer Treatment: A Narrative Review. Proc. Pak. Acad. Sci..

[42] Gutiérrez Coronado O., Sandoval Salazar C., Muñoz Carrillo J.L., Gutiérrez Villalobos O.A., Miranda Beltrán M.D.L.L., Soriano Hernández A.D., Beltrán Campos V., Villalobos Gutiérrez P.T. (2025). Functionalized Nanomaterials in Cancer Treatment: A Review. Int. J. Mol. Sci..

[43] Horn D., Rieger J. (2001). Organic Nanoparticles in the Aqueous Phase-Theory, Experiment, and Use. Angew. Chem. Int. Ed..

[44] Torchilin V.P. (2005). Recent Advances with Liposomes as Pharmaceutical Carriers. Nat. Rev. Drug Discov..

[45] Wissing S.A., Kayser O., Müller R.H. (2004). Solid Lipid Nanoparticles for Parenteral Drug Delivery. Adv. Drug Deliv. Rev..

[46] Koziara J.M., Lockman P.R., Allen D.D., Mumper R.J. (2004). Paclitaxel Nanoparticles for the Potential Treatment of Brain Tumors. J. Control. Release.

[47] Steiniger S.C.J., Kreuter J., Khalansky A.S., Skidan I.N., Bobruskin A.I., Smirnova Z.S., Severin S.E., Uhl R., Kock M., Geiger K.D. (2004). Chemotherapy of Glioblastoma in Rats Using Doxorubicin-Loaded Nanoparticles. Int. J. Cancer.

[48] Kou L., Bhutia Y.D., Yao Q., He Z., Sun J., Ganapathy V. (2018). Transporter-Guided Delivery of Nanoparticles to Improve Drug Permeation across Cellular Barriers and Drug Exposure to Selective Cell Types. Front. Pharmacol..

[49] Lammers T., Hennink W.E., Storm G. (2008). Tumour-Targeted Nanomedicines: Principles and Practice. Br. J. Cancer.

[50] Danhier F., Feron O., Préat V. (2010). To Exploit the Tumor Microenvironment: Passive and Active Tumor Targeting of Nanocarriers for Anti-Cancer Drug Delivery. J. Control. Release.

[51] Byrne J.D., Betancourt T., Brannon-Peppas L. (2008). Active Targeting Schemes for Nanoparticle Systems in Cancer Therapeutics. Adv. Drug Deliv. Rev..

[52] Maruyama K. (2011). Intracellular Targeting Delivery of Liposomal Drugs to Solid Tumors Based on EPR Effects. Adv. Drug Deliv. Rev..

[53] Muthu M.S., Singh S. (2009). Targeted Nanomedicines: Effective Treatment Modalities for Cancer, AIDS and Brain Disorders. Nanomedicine.

[54] Kataoka K., Harada A., Nagasaki Y. (2001). Block Copolymer Micelles for Drug Delivery: Design, Characterization and Biological Significance. Adv. Drug Deliv. Rev..

[55] Soppimath K.S., Aminabhavi T.M., Kulkarni A.R., Rudzinski W.E. (2001). Biodegradable Polymeric Nanoparticles as Drug Delivery Devices. J. Control. Release Off. J. Control. Release Soc..

[56] Allen T.M., Cullis P.R. (2013). Liposomal Drug Delivery Systems: From Concept to Clinical Applications. Adv. Drug Deliv. Rev..

[57] Gillies E.R., Fréchet J.M.J. (2005). Dendrimers and Dendritic Polymers in Drug Delivery. Drug Discov. Today.

[58] Ahn B., Park J., Singha K., Park H., Kim W.J. (2013). Mesoporous Silica Nanoparticle-Based Cisplatin Prodrug Delivery and Anticancer Effect under Reductive Cellular Environment. J. Mater. Chem. B.

[59] Doane T.L., Burda C. (2012). The Unique Role of Nanoparticles in Nanomedicine: Imaging, Drug Delivery and Therapy. Chem. Soc. Rev..

[60] Navya P.N., Kaphle A., Srinivas S.P., Bhargava S.K., Rotello V.M., Daima H.K. (2019). Current Trends and Challenges in Cancer Management and Therapy Using Designer Nanomaterials. Nano Converg..

[61] Ambrogio M.W., Thomas C.R., Zhao Y.-L., Zink J.I., Stoddart J.F. (2011). Mechanized Silica Nanoparticles: A New Frontier in Theranostic Nanomedicine. Acc. Chem. Res..

[62] Singh R., Sharma A., Saji J., Umapathi A., Kumar S., Daima H.K. (2022). Smart Nanomaterials for Cancer Diagnosis and Treatment. Nano Converg..

[63] Shi J., Kantoff P.W., Wooster R., Farokhzad O.C. (2017). Cancer Nanomedicine: Progress, Challenges and Opportunities. Nat. Rev. Cancer.

[64] Imantay A., Mashurov N., Zhaisanbayeva B.A., Mun E.A. (2025). Doxorubicin-Conjugated Nanoparticles for Potential Use as Drug Delivery Systems. Nanomaterials.

[65] Lancet J.E., Uy G.L., Cortes J.E., Newell L.F., Lin T.L., Ritchie E.K., Stuart R.K., Strickland S.A., Hogge D., Solomon S.R. (2016). Final Results of a Phase III Randomized Trial of CPX-351 versus 7+3 in Older Patients with Newly Diagnosed High Risk (Secondary) AML. J. Clin. Oncol..

[66] Vagena I.-A., Malapani C., Gatou M.-A., Lagopati N., Pavlatou E.A. (2025). Enhancement of EPR Effect for Passive Tumor Targeting: Current Status and Future Perspectives. Appl. Sci..

[67] Mahmoud A.M., Deambrogi C. (2025). Advancements in Nanotechnology for Targeted and Controlled Drug Delivery in Hematologic Malignancies: Shaping the Future of Targeted Therapeutics. Appl. Biosci..

[68] Casini A., Pöthig A. (2024). Metals in Cancer Research: Beyond Platinum Metallodrugs. ACS Cent. Sci..

[69] Ulbrich K., Holá K., Šubr V., Bakandritsos A., Tuček J., Zbořil R. (2016). Targeted Drug Delivery with Polymers and Magnetic Nanoparticles: Covalent and Noncovalent Approaches, Release Control, and Clinical Studies. Chem. Rev..

[70] Muhamad N., Plengsuriyakarn T., Na-bangchang K. (2018). Application of Active Targeting Nanoparticle Delivery System for Chemotherapeutic Drugs and Traditional/Herbal Medicines in Cancer Therapy: A Systematic Review. Int. J. Nanomed..

[71] Pearce A.K., O’Reilly R.K. (2019). Insights into Active Targeting of Nanoparticles in Drug Delivery: Advances in Clinical Studies and Design Considerations for Cancer Nanomedicine. Bioconjugate Chem..

[72] Rafati N., Zarepour A., Bigham A., Khosravi A., Naderi-Manesh H., Iravani S., Zarrabi A. (2024). Nanosystems for Targeted Drug Delivery: Innovations and Challenges in Overcoming the Blood-Brain Barrier for Neurodegenerative Disease and Cancer Therapy. Int. J. Pharm..

[73] Wang X., Guo Z. (2012). Targeting and Delivery of Platinum-Based Anticancer Drugs. Chem. Soc. Rev..

[74] Cheng X., Xie Q., Sun Y. (2023). Advances in Nanomaterial-Based Targeted Drug Delivery Systems. Front. Bioeng. Biotechnol..

[75] Tannock I.F., Rotin D. (1989). Acid pH in Tumors and Its Potential for Therapeutic Exploitation. Cancer Res..

[76] Storr T., Thompson K.H., Orvig C. (2006). Design of Targeting Ligands in Medicinal Inorganic Chemistry. Chem. Soc. Rev..

[77] Shaw R.J. (2006). Glucose Metabolism and Cancer. Curr. Opin. Cell Biol..

[78] Johnstone T.C., Suntharalingam K., Lippard S.J. (2016). The Next Generation of Platinum Drugs: Targeted Pt(II) Agents, Nanoparticle Delivery, and Pt(IV) Prodrugs. Chem. Rev..

[79] Hamanaka R.B., Chandel N.S. (2012). Targeting Glucose Metabolism for Cancer Therapy. J. Exp. Med..

[80] Xiao Y.-F., Jie M.-M., Li B.-S., Hu C.-J., Xie R., Tang B., Yang S.-M. (2015). Peptide-Based Treatment: A Promising Cancer Therapy. J. Immunol. Res..

[81] Seroka B., Łotowski Z., Hryniewicka A., Rárová L., Sicinski R.R., Tomkiel A.M., Morzycki J.W. (2020). Synthesis of New Cisplatin Derivatives from Bile Acids. Molecules.

[82] Weitman S.D., Lark R.H., Coney L.R., Fort D.W., Frasca V., Zurawski V.R.J., Kamen B.A. (1992). Distribution of the Folate Receptor GP38 in Normal and Malignant Cell Lines and Tissues. Cancer Res..

[83] Sudimack J., Lee R.J. (2000). Targeted Drug Delivery via the Folate Receptor. Adv. Drug Deliv. Rev..

[84] Vitols K.S., Montejano Y., Duffy T., Pope L., Grundler G., Huennekens F.M. (1987). Platinum-Folate Compounds: Synthesis, Properties and Biological Activity. Adv. Enzym. Regul..

[85] Attarwala H. (2010). Role of Antibodies in Cancer Targeting. J. Nat. Sci. Biol. Med..

[86] Jin S., Sun Y., Liang X., Gu X., Ning J., Xu Y., Chen S., Pan L. (2022). Emerging New Therapeutic Antibody Derivatives for Cancer Treatment. Signal Transduct. Target. Ther..

[87] Targeted Antibodies: mAbs, ADCs, BiTEs, and More. https://www.cancerresearch.org/en-us/immunotherapy/treatment-types/targeted-antibodies.

[88] Haxton K.J., Burt H.M. (2009). Polymeric Drug Delivery of Platinum-Based Anticancer Agents. J. Pharm. Sci..

[89] Chan W.C.W., Maxwell D.J., Gao X., Bailey R.E., Han M., Nie S. (2002). Luminescent Quantum Dots for Multiplexed Biological Detection and Imaging. Curr. Opin. Biotechnol..

[90] Sanchez-Cano C., Hannon M.J. (2009). Novel and Emerging Approaches for the Delivery of Metallo-Drugs. Dalton Trans..

[91] Niculescu A.-G., Grumezescu A.M. (2022). Novel Tumor-Targeting Nanoparticles for Cancer Treatment—A Review. Int. J. Mol. Sci..

[92] Liu B., Duan H., Liu Z., Liu Y., Chu H. (2024). DNA-Functionalized Metal or Metal-Containing Nanoparticles for Biological Applications. Dalton Trans..

[93] Di Marco M., Shamsuddin S., Razak K.A., Aziz A.A., Devaux C., Borghi E., Levy L., Sadun C. (2010). Overview of the Main Methods Used to Combine Proteins with Nanosystems: Absorption, Bioconjugation, and Encapsulation. Int. J. Nanomed..

[94] Hong V., Presolski S.I., Ma C., Finn M.G. (2009). Analysis and Optimization of Copper-Catalyzed Azide-Alkyne Cycloaddition for Bioconjugation. Angew. Chem. Int. Ed. Engl..

[95] Prescher J.A., Bertozzi C.R. (2005). Chemistry in Living Systems. Nat. Chem. Biol..

[96] Blackman M.L., Royzen M., Fox J.M. (2008). Tetrazine Ligation: Fast Bioconjugation Based on Inverse-Electron-Demand Diels−Alder Reactivity. J. Am. Chem. Soc..

[97] Devaraj N.K., Upadhyay R., Haun J.B., Hilderbrand S.A., Weissleder R. (2009). Fast and Sensitive Pretargeted Labeling of Cancer Cells through a Tetrazine/Trans-Cyclooctene Cycloaddition. Angew. Chem. Int. Ed..

[98] Algar W.R., Prasuhn D.E., Stewart M.H., Jennings T.L., Blanco-Canosa J.B., Dawson P.E., Medintz I.L. (2011). The Controlled Display of Biomolecules on Nanoparticles: A Challenge Suited to Bioorthogonal Chemistry. Bioconjug. Chem..

[99] Gupta M., Caniard A., Touceda-Varela Á., Campopiano D.J., Mareque-Rivas J.C. (2008). Nitrilotriacetic Acid-Derivatized Quantum Dots for Simple Purification and Site-Selective Fluorescent Labeling of Active Proteins in a Single Step. Bioconjugate Chem..

[100] Torchilin V.P. (2006). Multifunctional Nanocarriers. Adv. Drug Deliv. Rev..

[101] Akbarzadeh A., Rezaei-Sadabady R., Davaran S., Joo S.W., Zarghami N., Hanifehpour Y., Samiei M., Kouhi M., Nejati-Koshki K. (2013). Liposome: Classification, Preparation, and Applications. Nanoscale Res. Lett..

[102] Sawant R.R., Torchilin V.P. (2010). Liposomes as ‘Smart’ Pharmaceutical Nanocarriers. Soft Matter.

[103] Çağdaş M., Sezer A.D., Bucak S., Sezer A.D. (2014). Liposomes as Potential Drug Carrier Systems for Drug Delivery. Application of Nanotechnology in Drug Delivery.

[104] Zielińska A., Carreiró F., Oliveira A.M., Neves A., Pires B., Venkatesh D.N., Durazzo A., Lucarini M., Eder P., Silva A.M. (2020). Polymeric Nanoparticles: Production, Characterization, Toxicology and Ecotoxicology. Molecules.

[105] Selvakumaran S., Muhamad I., Md Lazim N.A. (2014). Designing Polymeric Nanoparticles for Targeted Drug Delivery System. Nanomedicine.

[106] Eltaib L. (2025). Polymeric Nanoparticles in Targeted Drug Delivery: Unveiling the Impact of Polymer Characterization and Fabrication. Polymers.

[107] Bhardwaj H., Jangde R.K. (2023). Current Updated Review on Preparation of Polymeric Nanoparticles for Drug Delivery and Biomedical Applications. Next Nanotechnol..

[108] Larson N., Ghandehari H. (2012). Polymeric Conjugates for Drug Delivery. Chem. Mater..

[109] Kopeček J. (2013). Polymer-Drug Conjugates: Origins, Progress to Date and Future Directions. Adv. Drug Deliv. Rev..

[110] Duncan R. (2007). Designing Polymer Conjugates as Lysosomotropic Nanomedicines. Biochem. Soc. Trans..

[111] Satchi-Fainaro R., Duncan R., Barnes C.M. (2006). Polymer Therapeutics for Cancer: Current Status and Future Challenges. Polymer Therapeutics II.

[112] Park J.H., Lee S., Kim J.H., Park K., Kim K., Kwon I.C. (2008). Polymeric Nanomedicine for Cancer Therapy. Prog. Polym. Sci. (Oxf.).

[113] Kratz F., Müller I.A., Ryppa C., Warnecke A. (2008). Prodrug Strategies in Anticancer Chemotherapy. ChemMedChem.

[114] Venditto V.J., Szoka F.C. (2013). Cancer Nanomedicines: So Many Papers and so Few Drugs!. Adv. Drug Deliv. Rev..

[115] Canal F., Sanchis J., Vicent M.J. (2011). Polymer–Drug Conjugates as Nano-Sized Medicines. Curr. Opin. Biotechnol..

[116] Chauhan A.S. (2018). Dendrimers for Drug Delivery. Molecules.

[117] Laurent R., Maraval V., Bernardes-Génisson V., Caminade A.-M. (2024). Dendritic Pyridine–Imine Copper Complexes as Metallo-Drugs. Molecules.

[118] Singh J., Jain K., Mehra N.K., Jain N.K. (2016). Dendrimers in Anticancer Drug Delivery: Mechanism of Interaction of Drug and Dendrimers. Artif. Cells Nanomed. Biotechnol..

[119] Noriega-Luna B., Godínez L.A., Rodríguez F.J., Rodríguez A., Zaldívar-Lelo de Larrea G., Sosa-Ferreyra C.F., Mercado-Curiel R.F., Manríquez J., Bustos E. (2014). Applications of Dendrimers in Drug Delivery Agents, Diagnosis, Therapy, and Detection. J. Nanomater..

[120] Navath R.S., Menjoge A.R., Wang B., Romero R., Kannan S., Kannan R.M. (2010). Amino Acid-Functionalized Dendrimers with Heterobifunctional Chemoselective Peripheral Groups for Drug Delivery Applications. Biomacromolecules.

[121] Movliya V.R., Patel M.P. (2019). Role of Dendrimer in Drug Solubilization—A Review. Drug Deliv. Lett..

[122] Kesharwani P., Jain K., Jain N.K. (2014). Dendrimer as Nanocarrier for Drug Delivery. Prog. Polym. Sci..

[123] Yellepeddi V.K., Kumar A., Maher D.M., Chauhan S.C., Vangara K.K., Palakurthi S. (2011). Biotinylated PAMAM Dendrimers for Intracellular Delivery of Cisplatin to Ovarian Cancer: Role of SMVT. Anticancer Res..

[124] Qi R., Majoros I., Misra A.C., Koch A.E., Campbell P., Marotte H., Bergin I.L., Cao Z., Goonewardena S., Morry J. (2015). Folate Receptor-Targeted Dendrimer-Methotrexate Conjugate for Inflammatory Arthritis. J. Biomed. Nanotechnol..

[125] Szota M., Reczyńska-Kolman K., Pamuła E., Michel O., Kulbacka J., Jachimska B. (2021). Poly(Amidoamine) Dendrimers as Nanocarriers for 5-Fluorouracil: Effectiveness of Complex Formation and Cytotoxicity Studies. Int. J. Mol. Sci..

[126] Patra J.K., Das G., Fraceto L.F., Campos E.V.R., Rodriguez-Torres M.d.P., Acosta-Torres L.S., Diaz-Torres L.A., Grillo R., Swamy M.K., Sharma S. (2018). Nano Based Drug Delivery Systems: Recent Developments and Future Prospects. J. Nanobiotechnol..

[127] Seixas N., Ravanello B.B., Morgan I., Kaluđerović G.N., Wessjohann L.A. (2019). Chlorambucil Conjugated Ugi Dendrimers with PAMAM-NH_2_ Core and Evaluation of Their Anticancer Activity. Pharmaceutics.

[128] Oberoi H.S., Nukolova N.V., Kabanov A.V., Bronich T.K. (2013). Nanocarriers for Delivery of Platinum Anticancer Drugs. Adv. Drug Deliv. Rev..

[129] Vidu R., Rahman M., Mahmoudi M., Enachescu M., Poteca T., Opris I. (2014). Nanostructures: A Platform for Brain Repair and Augmentation. Front. Syst. Neurosci..

[130] Iijima S. (1991). Helical Microtubules of Graphitic Carbon. Nature.

[131] Liang F., Chen B. (2010). A Review on Biomedical Applications of Single-Walled Carbon Nanotubes. Curr. Med. Chem..

[132] Ajima K., Yudasaka M., Murakami T., Maigné A., Shiba K., Iijima S. (2005). Carbon Nanohorns as Anticancer Drug Carriers. Mol. Pharm..

[133] Feazell R.P., Nakayama-Ratchford N., Dai H., Lippard S.J. (2007). Soluble Single-Walled Carbon Nanotubes as Longboat Delivery Systems for Platinum(IV) Anticancer Drug Design. J. Am. Chem. Soc..

[134] Tripisciano C., Kraemer K., Taylor A., Borowiak-Palen E. (2009). Single-Wall Carbon Nanotubes Based Anticancer Drug Delivery System. Chem. Phys. Lett..

[135] Boncel S., Zając P., Koziol K.K.K. (2013). Liberation of Drugs from Multi-Wall Carbon Nanotube Carriers. J. Control..

[136] Sonowal L., Gautam S. (2024). Advancements and Challenges in Carbon Nanotube-Based Drug Delivery Systems. Nano-Struct. Nano-Objects.

[137] Li J., Yap S.Q., Chin C.F., Tian Q., Yoong S.L., Pastorin G., Ang W.H. (2012). Platinum(IV) Prodrugs Entrapped within Multiwalled Carbon Nanotubes: Selective Release by Chemical Reduction and Hydrophobicity Reversal. Chem. Sci..

[138] Ma P., Xiao H., Li C., Dai Y., Cheng Z., Hou Z., Lin J. (2015). Inorganic Nanocarriers for Platinum Drug Delivery. Mater. Today.

[139] Mekaru H., Lu J., Tamanoi F. (2015). Development of Mesoporous Silica-Based Nanoparticles with Controlled Release Capability for Cancer Therapy. Adv. Drug Deliv. Rev..

[140] Qin J.-X., Yang X.-G., Lv C.-F., Li Y.-Z., Liu K.-K., Zang J.-H., Yang X., Dong L., Shan C.-X. (2021). Nanodiamonds: Synthesis, Properties, and Applications in Nanomedicine. Mater. Des..

[141] Feinberg A. (2014). How These Microscopic Diamonds Are Going to Shape the Future. Gizmodo. https://gizmodo.com/how-these-microscopic-diamonds-are-going-to-shape-the-f-1459620387.

[142] Murphy C.J., Gole A.M., Stone J.W., Sisco P.N., Alkilany A.M., Goldsmith E.C., Baxter S.C. (2008). Gold Nanoparticles in Biology: Beyond Toxicity to Cellular Imaging. Acc. Chem. Res..

[143] Szewczyk O.K., Roszczenko P., Czarnomysy R., Bielawska A., Bielawski K. (2022). An Overview of the Importance of Transition-Metal Nanoparticles in Cancer Research. Int. J. Mol. Sci..

[144] Bohren C.F., Huffman D.R. (1983). Absorption and Scattering of Light by Small Particles.

[145] Hirsch L.R., Stafford R.J., Bankson J.A., Sershen S.R., Rivera B., Price R.E., Hazle J.D., Halas N.J., West J.L. (2003). Nanoshell-Mediated near-Infrared Thermal Therapy of Tumors under Magnetic Resonance Guidance. Proc. Natl. Acad. Sci. USA.

[146] Na H.B., Song I.C., Hyeon T. (2009). Inorganic Nanoparticles for MRI Contrast Agents. Adv. Mater..

[147] Laurent S., Forge D., Port M., Roch A., Robic C., Vander Elst L., Muller R.N. (2008). Magnetic Iron Oxide Nanoparticles: Synthesis, Stabilization, Vectorization, Physicochemical Characterizations, and Biological Applications. Chem. Rev..

[148] Hao R., Xing R., Xu Z., Hou Y., Gao S., Sun S. (2010). Synthesis, Functionalization, and Biomedical Applications of Multifunctional Magnetic Nanoparticles. Adv. Mater..

[149] McBain S.C., Yiu H.H.P., Dobson J. (2008). Magnetic Nanoparticles for Gene and Drug Delivery. Int. J. Nanomed..

[150] Păduraru D.N., Ion D., Niculescu A.-G., Mușat F., Andronic O., Grumezescu A.M., Bolocan A. (2022). Recent Developments in Metallic Nanomaterials for Cancer Therapy, Diagnosing and Imaging Applications. Pharmaceutics.

[151] Gao J., Gu H., Xu B. (2009). Multifunctional Magnetic Nanoparticles: Design, Synthesis, and Biomedical Applications. Acc. Chem. Res..

[152] Veiseh O., Gunn J.W., Zhang M. (2010). Design and Fabrication of Magnetic Nanoparticles for Targeted Drug Delivery and Imaging. Adv. Drug Deliv. Rev..

[153] Pankhurst Q.A., Connolly J., Jones S.K., Dobson J. (2003). Applications of Magnetic Nanoparticles in Biomedicine. J. Phys. D Appl. Phys..

[154] Hernandez R., Tseng H.-R., Wong J.W., Stoddart J.F., Zink J.I. (2004). An Operational Supramolecular Nanovalve. J. Am. Chem. Soc..

[155] Lee C.-H., Cheng S.-H., Huang I.-P., Souris J.S., Yang C.-S., Mou C.-Y., Lo L.-W. (2010). Intracellular pH-Responsive Mesoporous Silica Nanoparticles for the Controlled Release of Anticancer Chemotherapeutics. Angew. Chem. Int. Ed..

[156] Hwang A.A., Lu J., Tamanoi F., Zink J.I. (2015). Functional Nanovalves on Protein-Coated Nanoparticles for In Vitro and In Vivo Controlled Drug Delivery. Small.

[157] Meng H., Xue M., Xia T., Zhao Y.-L., Tamanoi F., Stoddart J.F., Zink J.I., Nel A.E. (2010). Autonomous in Vitro Anticancer Drug Release from Mesoporous Silica Nanoparticles by pH-Sensitive Nanovalves. J. Am. Chem. Soc..

[158] Park C., Oh K., Lee S.C., Kim C. (2007). Controlled Release of Guest Molecules from Mesoporous Silica Particles Based on a pH-Responsive Polypseudorotaxane Motif. Angew. Chem. Int. Ed..

[159] Gan Q., Lu X., Yuan Y., Qian J., Zhou H., Lu X., Shi J., Liu C. (2011). A Magnetic, Reversible pH-Responsive Nanogated Ensemble Based on Fe3O4 Nanoparticles-Capped Mesoporous Silica. Biomaterials.

[160] Aznar E., Marcos M.D., Martínez-Máñez R., Sancenón F., Soto J., Amorós P., Guillem C. (2009). pH- and Photo-Switched Release of Guest Molecules from Mesoporous Silica Supports. J. Am. Chem. Soc..

[161] Gao C., Zheng H., Xing L., Shu M., Che S. (2010). Designable Coordination Bonding in Mesopores as a pH-Responsive Release System. Chem. Mater..

[162] Popat A., Liu J., Lu G.Q., Qiao S.Z. (2012). A pH-Responsive Drug Delivery System Based on Chitosan Coated Mesoporous Silica Nanoparticles. J. Mater. Chem..

[163] Chen F., Zhu Y. (2012). Chitosan Enclosed Mesoporous Silica Nanoparticles as Drug Nano-Carriers: Sensitive Response to the Narrow pH Range. Microporous Mesoporous Mater..

[164] Ashley C.E., Carnes E.C., Phillips G.K., Padilla D., Durfee P.N., Brown P.A., Hanna T.N., Liu J., Phillips B., Carter M.B. (2011). The Targeted Delivery of Multicomponent Cargos to Cancer Cells by Nanoporous Particle-Supported Lipid Bilayers. Nat. Mater..

[165] Théron C., Gallud A., Carcel C., Gary-Bobo M., Maynadier M., Garcia M., Lu J., Tamanoi F., Zink J.I., Wong Chi Man M. (2014). Hybrid Mesoporous Silica Nanoparticles with pH-Operated and Complementary H-Bonding Caps as an Autonomous Drug-Delivery System. Chemistry.

[166] Dong J., Xue M., Zink J.I. (2013). Functioning of Nanovalves on Polymer Coated Mesoporous Silica Nanoparticles. Nanoscale.

[167] Ambrogio M.W., Pecorelli T.A., Patel K., Khashab N.M., Trabolsi A., Khatib H.A., Botros Y.Y., Zink J.I., Stoddart J.F. (2010). Snap-Top Nanocarriers. Org. Lett..

[168] Lai C.-Y., Trewyn B.G., Jeftinija D.M., Jeftinija K., Xu S., Jeftinija S., Lin V.S.-Y. (2003). A Mesoporous Silica Nanosphere-Based Carrier System with Chemically Removable CdS Nanoparticle Caps for Stimuli-Responsive Controlled Release of Neurotransmitters and Drug Molecules. J. Am. Chem. Soc..

[169] Giri S., Trewyn B.G., Stellmaker M.P., Lin V.S.-Y. (2005). Stimuli-Responsive Controlled-Release Delivery System Based on Mesoporous Silica Nanorods Capped with Magnetic Nanoparticles. Angew. Chem. Int. Ed..

[170] Zhang Q., Liu F., Nguyen K.T., Ma X., Wang X., Xing B., Zhao Y. (2012). Multifunctional Mesoporous Silica Nanoparticles for Cancer-Targeted and Controlled Drug Delivery. Adv. Funct. Mater..

[171] Kim H., Kim S., Park C., Lee H., Park H.J., Kim C. (2010). Glutathione-Induced Intracellular Release of Guests from Mesoporous Silica Nanocontainers with Cyclodextrin Gatekeepers. Adv. Mater..

[172] Liu R., Zhao X., Wu T., Feng P. (2008). Tunable Redox-Responsive Hybrid Nanogated Ensembles. J. Am. Chem. Soc..

[173] Luo Z., Cai K., Hu Y., Zhao L., Liu P., Duan L., Yang W. (2011). Mesoporous Silica Nanoparticles End-Capped with Collagen: Redox-Responsive Nanoreservoirs for Targeted Drug Delivery. Angew. Chem. Int. Ed..

[174] Lu J., Choi E., Tamanoi F., Zink J.I. (2008). Light-Activated Nanoimpeller-Controlled Drug Release in Cancer Cells. Small.

[175] Ferris D.P., Zhao Y.-L., Khashab N.M., Khatib H.A., Stoddart J.F., Zink J.I. (2009). Light-Operated Mechanized Nanoparticles. J. Am. Chem. Soc..

[176] Tarn D., Ferris D.P., Barnes J.C., Ambrogio M.W., Stoddart J.F., Zink J.I. (2014). A Reversible Light-Operated Nanovalve on Mesoporous Silica Nanoparticles. Nanoscale.

[177] Croissant J., Maynadier M., Gallud A., N’Dongo H.P., Nyalosaso J.L., Derrien G., Charnay C., Durand J.-O., Raehm L., Serein-Spirau F. (2013). Two-Photon-Triggered Drug Delivery in Cancer Cells Using Nanoimpellers. Angew. Chem. Int. Ed..

[178] Croissant J., Chaix A., Mongin O., Wang M., Clément S., Raehm L., Durand J.-O., Hugues V., Blanchard-Desce M., Maynadier M. (2014). Two-Photon-Triggered Drug Delivery via Fluorescent Nanovalves. Small.

[179] Guardado-Alvarez T.M., Sudha Devi L., Russell M.M., Schwartz B.J., Zink J.I. (2013). Activation of Snap-Top Capped Mesoporous Silica Nanocontainers Using Two near-Infrared Photons. J. Am. Chem. Soc..

[180] Lin Q., Huang Q., Li C., Bao C., Liu Z., Li F., Zhu L. (2010). Anticancer Drug Release from a Mesoporous Silica Based Nanophotocage Regulated by Either a One- or Two-Photon Process. J. Am. Chem. Soc..

[181] Liong M., Lu J., Kovochich M., Xia T., Ruehm S.G., Nel A.E., Tamanoi F., Zink J.I. (2008). Multifunctional Inorganic Nanoparticles for Imaging, Targeting, and Drug Delivery. ACS Nano.

[182] Saint-Cricq P., Deshayes S., Zink J.I., Kasko A.M. (2015). Magnetic Field Activated Drug Delivery Using Thermodegradable Azo-Functionalised PEG-Coated Core–Shell Mesoporous Silica Nanoparticles. Nanoscale.

[183] Baeza A., Guisasola E., Ruiz-Hernández E., Vallet-Regí M. (2012). Magnetically Triggered Multidrug Release by Hybrid Mesoporous Silica Nanoparticles. Chem. Mater..

[184] Chen P.-J., Hu S.-H., Hsiao C.-S., Chen Y.-Y., Liu D.-M., Chen S.-Y. (2011). Multifunctional Magnetically Removable Nanogated Lids of Fe_3_O_4_–Capped Mesoporous Silica Nanoparticles for Intracellular Controlled Release and MR Imaging. J. Mater. Chem..

[185] Hom C., Lu J., Liong M., Luo H., Li Z., Zink J.I., Tamanoi F. (2010). Mesoporous Silica Nanoparticles Facilitate Delivery of siRNA to Shutdown Signaling Pathways in Mammalian Cells. Small.

[186] Xie X., Yue T., Gu W., Cheng W., He L., Ren W., Li F., Piao J.-G. (2023). Recent Advances in Mesoporous Silica Nanoparticles Delivering siRNA for Cancer Treatment. Pharmaceutics.

[187] Finlay J., Roberts C.M., Dong J., Zink J.I., Tamanoi F., Glackin C.A. (2015). Mesoporous Silica Nanoparticle Delivery of Chemically Modified siRNA against TWIST1 Leads to Reduced Tumor Burden. Nanomed. Nanotechnol. Biol. Med..

[188] Krajnović T., Pantelić N.Đ., Wolf K., Eichhorn T., Maksimović-Ivanić D., Mijatović S., Wessjohann L.A., Kaluđerović G.N. (2022). Anticancer Potential of Xanthohumol and Isoxanthohumol Loaded into SBA-15 Mesoporous Silica Particles against B16F10 Melanoma Cells. Materials.

[189] Jänicke P., Lennicke C., Meister A., Seliger B., Wessjohann L.A., Kaluđerović G.N. (2021). Fluorescent Spherical Mesoporous Silica Nanoparticles Loaded with Emodin: Synthesis, Cellular Uptake and Anticancer Activity. Mater. Sci. Eng. C.

[190] Krajnović T., Maksimović-Ivanić D., Mijatović S., Drača D., Wolf K., Edeler D., Wessjohann L.A., Kaluđerović G.N. (2018). Drug Delivery System for Emodin Based on Mesoporous Silica SBA-15. Nanomaterials.

[191] Jankovic-Tomanic M., Todorovic D., Stanivukovic Z., Peric Mataruga V., Wessjohann L.A., Kaluđerović G.N. (2017). Mesoporous Silica Nanoparticles SBA-15 Loaded with Emodin Upregulate the Antioxidative Defense of *Euproctis chrysorrhoea* (L.) Larvae. Turk. J. Biol..

[192] Kopecek J., Kopecková P., Minko T., Lu Z. (2000). HPMA Copolymer-Anticancer Drug Conjugates: Design, Activity, and Mechanism of Action. Eur. J. Pharm. Biopharm..

[193] Kabanov A.V., Bronich T.K., Kabanov V.A., Yu K., Eisenberg A. (1996). Soluble Stoichiometric Complexes from Poly(N-Ethyl-4-Vinylpyridinium) Cations and Poly(Ethylene Oxide)-Block-Polymethacrylate Anions. Macromolecules.

[194] Nishiyama N., Yokoyama M., Aoyagi T., Okano T., Sakurai Y., Kataoka K. (1999). Preparation and Characterization of Self-Assembled Polymer−Metal Complex Micelle from Cis-Dichlorodiammineplatinum(II) and Poly(Ethylene Glycol)−Poly(α,β-Aspartic Acid) Block Copolymer in an Aqueous Medium. Langmuir.

[195] Zhang Z., Chen J., Wen T., Deng H., Zhang Y., Guo H., Chang H., Xu H., Zhang W. (2025). Quantification of Cisplatin Encapsulated in Nanomedicine: An Overview. Biosensors.

[196] Rice J.R., Gerberich J.L., Nowotnik D.P., Howell S.B. (2006). Preclinical Efficacy and Pharmacokinetics of AP5346, a Novel Diaminocyclohexane-Platinum Tumor-Targeting Drug Delivery System. Clin. Cancer Res..

[197] Sood P., Thurmond K.B., Jacob J.E., Waller L.K., Silva G.O., Stewart D.R., Nowotnik D.P. (2006). Synthesis and Characterization of AP5346, a Novel Polymer-Linked Diaminocyclohexyl Platinum Chemotherapeutic Agent. Bioconjugate Chem..

[198] Oberoi H.S., Nukolova N.V., Zhao Y., Cohen S.M., Kabanov A.V., Bronich T.K. (2012). Preparation and In Vivo Evaluation of Dichloro(1,2-Diaminocyclohexane)Platinum(II)-Loaded Core Cross-Linked Polymer Micelles. Chemother. Res. Pract..

[199] Howell B.A., Fan D. (2010). Poly(Amidoamine) Dendrimer-Supported Organoplatinum Antitumour Agents. Proc. R. Soc. Lond. A Math. Phys. Eng. Sci..

[200] Haririan I., Alavidjeh M.S., Khorramizadeh M.R., Ardestani M.S., Ghane Z.Z., Namazi H. (2010). Anionic Linear-Globular Dendrimer-Cis-Platinum (II) Conjugates Promote Cytotoxicity in Vitro against Different Cancer Cell Lines. Int. J. Nanomed..

[201] Kapp T., Dullin A., Gust R. (2010). Platinum(II)−Dendrimer Conjugates: Synthesis and Investigations on Cytotoxicity, Cellular Distribution, Platinum Release, DNA, and Protein Binding. Bioconjugate Chem..

[202] Ganpisetti R., Giridharan S., Vaskuri G.S.S.J., Narang N., Basim P., Dokmeci M.R., Ermis M., Rojekar S., Gholap A.D., Kommineni N. (2025). Biological Nanocarriers in Cancer Therapy: Cutting Edge Innovations in Precision Drug Delivery. Biomolecules.

[203] Boulikas T. (2004). Low Toxicity and Anticancer Activity of a Novel Liposomal Cisplatin (Lipoplatin) in Mouse Xenografts. Oncol. Rep..

[204] Murakami M., Cabral H., Matsumoto Y., Wu S., Kano M.R., Yamori T., Nishiyama N., Kataoka K. (2011). Improving Drug Potency and Efficacy by Nanocarrier-Mediated Subcellular Targeting. Sci. Transl. Med..

[205] Giljohann D.A., Seferos D.S., Daniel W.L., Massich M.D., Patel P.C., Mirkin C.A. (2010). Gold Nanoparticles for Biology and Medicine. Angew. Chem. Int. Ed..

[206] Brown S.D., Nativo P., Smith J.-A., Stirling D., Edwards P.R., Venugopal B., Flint D.J., Plumb J.A., Graham D., Wheate N.J. (2010). Gold Nanoparticles for the Improved Anticancer Drug Delivery of the Active Component of Oxaliplatin. J. Am. Chem. Soc..

[207] Wagstaff A.J., Brown S.D., Holden M.R., Craig G.E., Plumb J.A., Brown R.E., Schreiter N., Chrzanowski W., Wheate N.J. (2012). Cisplatin Drug Delivery Using Gold-Coated Iron Oxide Nanoparticles for Enhanced Tumour Targeting with External Magnetic Fields. Inorg. Chim. Acta.

[208] Craig G.E., Brown S.D., Lamprou D.A., Graham D., Wheate N.J. (2012). Cisplatin-Tethered Gold Nanoparticles That Exhibit Enhanced Reproducibility, Drug Loading, and Stability: A Step Closer to Pharmaceutical Approval?. Inorg. Chem..

[209] Dhar S., Daniel W.L., Giljohann D.A., Mirkin C.A., Lippard S.J. (2009). Polyvalent Oligonucleotide Gold Nanoparticle Conjugates as Delivery Vehicles for Platinum(IV) Warheads. J. Am. Chem. Soc..

[210] Min Y., Mao C.-Q., Chen S., Ma G., Wang J., Liu Y. (2012). Combating the Drug Resistance of Cisplatin Using a Platinum Prodrug Based Delivery System. Angew. Chem. Int. Ed..

[211] Singh A., Sahoo S.K. (2014). Magnetic Nanoparticles: A Novel Platform for Cancer Theranostics. Drug Discov. Today.

[212] Timerbaev A.R. (2022). Analytical Methodology for Developing Nanomaterials Designed for Magnetically-Guided Delivery of Platinum Anticancer Drugs. Talanta.

[213] Cheng K., Peng S., Xu C., Sun S. (2009). Porous Hollow Fe_3_O_4_ Nanoparticles for Targeted Delivery and Controlled Release of Cisplatin. J. Am. Chem. Soc..

[214] Xu C., Wang B., Sun S. (2009). Dumbbell-like Au−Fe3O4 Nanoparticles for Target-Specific Platin Delivery. J. Am. Chem. Soc..

[215] Xing R., Wang X., Zhang C., Wang J., Zhang Y., Song Y., Guo Z. (2011). Superparamagnetic Magnetite Nanocrystal Clusters as Potential Magnetic Carriers for the Delivery of Platinum Anticancer Drugs. J. Mater. Chem..

[216] Cheng Z., Dai Y., Kang X., Li C., Huang S., Lian H., Hou Z., Ma P., Lin J. (2014). Gelatin-Encapsulated Iron Oxide Nanoparticles for Platinum(IV) Prodrug Delivery, Enzyme-Stimulated Release and MRI. Biomaterials.

[217] Mamaeva V., Sahlgren C., Lindén M. (2013). Mesoporous Silica Nanoparticles in Medicine–Recent Advances. Adv. Drug Deliv. Rev..

[218] Santhamoorthy M., Asaithambi P., Ramkumar V., Elangovan N., Perumal I., Kim S.C. (2025). A Review on the Recent Advancements of Polymer-Modified Mesoporous Silica Nanoparticles for Drug Delivery Under Stimuli-Trigger. Polymers.

[219] Wang D., Xu Z., Chen Z., Liu X., Hou C., Zhang X., Zhang H. (2014). Fabrication of Single-Hole Glutathione-Responsive Degradable Hollow Silica Nanoparticles for Drug Delivery. ACS Appl. Mater. Interfaces.

[220] Li D., Zhang Y., Jin S., Guo J., Gao H., Wang C. (2014). Development of a Redox/pH Dual Stimuli-Responsive MSP@P(MAA-Cy) Drug Delivery System for Programmed Release of Anticancer Drugs in Tumour Cells. J. Mater. Chem. B.

[221] Lin C.-H., Cheng S.-H., Liao W.-N., Wei P.-R., Sung P.-J., Weng C.-F., Lee C.-H. (2012). Mesoporous Silica Nanoparticles for the Improved Anticancer Efficacy of Cis-Platin. Int. J. Pharm..

[222] He H., Xiao H., Kuang H., Xie Z., Chen X., Jing X., Huang Y. (2014). Synthesis of Mesoporous Silica Nanoparticle–Oxaliplatin Conjugates for Improved Anticancer Drug Delivery. Colloids Surf. B Biointerfaces.

[223] Tao Z., Xie Y., Goodisman J., Asefa T. (2010). Isomer-Dependent Adsorption and Release of Cis- and Trans-Platin Anticancer Drugs by Mesoporous Silica Nanoparticles. Langmuir.

[224] Tao Z., Toms B., Goodisman J., Asefa T. (2010). Mesoporous Silica Microparticles Enhance the Cytotoxicity of Anticancer Platinum Drugs. ACS Nano.

[225] Drača D., Edeler D., Saoud M., Dojčinović B., Dunđerović D., Đmura G., Maksimović-Ivanić D., Mijatović S., Kaluđerović G.N. (2021). Antitumor Potential of Cisplatin Loaded into SBA-15 Mesoporous Silica Nanoparticles against B16F1 Melanoma Cells: In Vitro and in Vivo Studies. J. Inorg. Biochem..

[226] Edeler D., Kaluđerović M.R., Dojčinović B., Schmidt H., Kaluđerović G.N. (2016). SBA-15 Mesoporous Silica Particles Loaded with Cisplatin Induce Senescence in B16F10 Cells. RSC Adv..

[227] Predarska I., Saoud M., Morgan I., Eichhorn T., Kaluđerović G.N., Hey-Hawkins E. (2022). Cisplatin−cyclooxygenase Inhibitor Conjugates, Free and Immobilised in Mesoporous Silica SBA-15, Prove Highly Potent against Triple-Negative MDA-MB-468 Breast Cancer Cell Line. Dalton Trans..

[228] Predarska I., Saoud M., Drača D., Morgan I., Komazec T., Eichhorn T., Mihajlović E., Dunđerović D., Mijatović S., Maksimović-Ivanić D. (2022). Mesoporous Silica Nanoparticles Enhance the Anticancer Efficacy of Platinum(IV)-Phenolate Conjugates in Breast Cancer Cell Lines. Nanomaterials.

[229] Balbín A., Gaballo F., Ceballos-Torres J., Prashar S., Fajardo M., Kaluđerović G.N., Gómez-Ruiz S. (2014). Dual Application of Pd Nanoparticles Supported on Mesoporous Silica SBA-15 and MSU-2: Supported Catalysts for C–C Coupling Reactions and Cytotoxic Agents against Human Cancer Cell Lines. RSC Adv..

[230] Edeler D., Arlt S., Petković V., Ludwig G., Drača D., Maksimović-Ivanić D., Mijatović S., Kaluđerović G.N. (2018). Delivery of [Ru(η^6^-*p*-Cymene)Cl_2_Ph_2_P(CH_2_)_3_SPh-κ*P*] Using Unfunctionalized and Mercapto Functionalized SBA-15 Mesoporous Silica: Preparation, Characterization and in Vitro Study. J. Inorg. Biochem..

[231] Mladenović M., Morgan I., Ilić N., Saoud M., Pergal M.V., Kaluđerović G.N., Knežević N.Ž. (2021). pH-Responsive Release of Ruthenium Metallotherapeutics from Mesoporous Silica-Based Nanocarriers. Pharmaceutics.

[232] Ellahioui Y., Patra M., Mari C., Kaabi R., Karges J., Gasser G., Gómez-Ruiz S. (2019). Mesoporous Silica Nanoparticles Functionalised with a Photoactive Ruthenium(II) Complex: Exploring the Formulation of a Metal-Based Photodynamic Therapy Photosensitiser. Dalton Trans..

[233] Pérez-Quintanilla D., Gómez-Ruiz S., Žižak Ž., Sierra I., Prashar S., del Hierro I., Fajardo M., Juranić Z.D., Kaluđerović G.N. (2009). A New Generation of Anticancer Drugs: Mesoporous Materials Modified with Titanocene Complexes. Chem. Eur. J..

[234] Caruso F., Rossi M. (2004). Antitumor Titanium Compounds. Mini Rev. Med. Chem..

[235] del Hierro I., Gómez-Ruiz S., Pérez Y., Cruz P., Prashar S., Fajardo M. (2018). Mesoporous SBA-15 Modified with Titanocene Complexes and Ionic Liquids: Interactions with DNA and Other Molecules of Biological Interest Studied by Solid State Electrochemical Techniques. Dalton Trans..

[236] Díaz-García D., Cenariu D., Pérez Y., Cruz P., del Hierro I., Prashar S., Fischer-Fodor E., Gómez-Ruiz S. (2018). Modulation of the Mechanism of Apoptosis in Cancer Cell Lines by Treatment with Silica-Based Nanostructured Materials Functionalized with Different Metallodrugs. Dalton Trans..

[237] Bulatović M.Z., Maksimović-Ivanić D., Bensing C., Gómez-Ruiz S., Steinborn D., Schmidt H., Mojić M., Korać A., Golić I., Pérez-Quintanilla D. (2014). Organotin(IV)-Loaded Mesoporous Silica as a Biocompatible Strategy in Cancer Treatment. Angew. Chem. Int. Ed..

[238] Edeler D., Drača D., Petković V., Natalio F., Maksimović-Ivanić D., Mijatović S., Schmidt H., Kaluđerović G.N. (2019). Impact of the Mesoporous Silica SBA-15 Functionalization on the Mode of Action of Ph_3_Sn(CH_2_)_6_OH. Mater. Sci. Eng. C.

[239] Maksimović-Ivanić D., Bulatović M., Edeler D., Bensing C., Golić I., Korać A., Kaluđerović G.N., Mijatović S. (2019). The Interaction between SBA-15 Derivative Loaded with Ph_3_Sn(CH_2_)_6_OH and Human Melanoma A375 Cell Line: Uptake and Stem Phenotype Loss. J. Biol. Inorg. Chem..

[240] Bensing C., Mojić M., Gómez-Ruiz S., Carralero S., Dojčinović B., Maksimović-Ivanić D., Mijatović S., Kaluđerović G.N. (2016). Evaluation of Functionalized Mesoporous Silica SBA-15 as a Carrier System for Ph_3_Sn(CH_2_)_3_OH against the A2780 Ovarian Carcinoma Cell Line. Dalton Trans..

[241] Ovejero-Paredes K., Díaz-García D., Mena-Palomo I., Marciello M., Lozano-Chamizo L., Morato Y.L., Prashar S., Gómez-Ruiz S., Filice M. (2022). Synthesis of a Theranostic Platform Based on Fibrous Silica Nanoparticles for the Enhanced Treatment of Triple-Negative Breast Cancer Promoted by a Combination of Chemotherapeutic Agents. Biomater. Adv..

[242] Choudante P.C., Nethi S.K., Díaz-García D., Prashar S., Misra S., Gómez-Ruiz S., Patra C.R. (2022). Tin-Loaded Mesoporous Silica Nanoparticles: Antineoplastic Properties and Genotoxicity Assessment. Biomater. Adv..

[243] Díaz-García D., Fischer-Fodor E., Vlad C.I., Méndez-Arriaga J.M., Prashar S., Gómez-Ruiz S. (2021). Study of Cancer Cell Cytotoxicity, Internalization and Modulation of Growth Factors Induced by Transferrin-Conjugated Formulations of Metallodrug-Functionalized Mesoporous Silica Nanoparticles. Microporous Mesoporous Mater..

